# Single-cell directional sensing at ultra-low chemoattractant concentrations from extreme first-passage events

**DOI:** 10.1007/s00285-026-02478-z

**Published:** 2026-10-29

**Authors:** Vincent Fiorino, Sean D. Lawley, Alan E. Lindsay

**Affiliations:** 1 Department of Applied and Computational Mathematics and Statistics, University of Notre Dame, Notre Dame, IN 46656 USA; 2 Department of Mathematics, University of Utah, Salt Lake City, UT 84112 USA

**Keywords:** Cell motility, Directional sensing, First passage times, Extreme statistics, 60G70, 62G32, 62P10, 92C17

## Abstract

We investigate single-cell directional sensing from diffusing chemoattractant signals released by a localized source. We focus on the low-concentration regime in which receptor activity is discrete and cellular decisions are made on timescales far shorter than those required for steady-state concentration profiles or receptor saturation to emerge. We derive analytic expressions for the joint distribution of receptor binding times and binding locations, conditional on the position of the source. We show that early binding events carry disproportionately more information about source directionality than later arrivals. Motivated by this observation, we propose and analyze several source-localization estimates that exploit early receptor binding statistics. For a directional estimate formed from an equally weighted average of the earliest impacts, we demonstrate that this estimate is accurate even from a small number of impacts, and derive an expression for the number of binding events that minimizes the mean squared error. Our results demonstrate that cells possess sufficient information to rapidly and accurately infer the directionality of sparse signals from a localized source through a small number of discrete receptor engagements, well before a steady-state gradient has been established.

## Introduction

The aim of this paper is to explore the theoretical limits by which cells can establish the directionality of chemical cues at ultra-low chemoattractant concentrations. A key cellular function is the ability to rapidly and accurately interpret the directionality of chemical or electric gradients to inform decisions on where to move, when to divide or whether to initiate a response to a threat (Copos et al. 2025; Henderson et al. 2019; Parent and Devreotes 1999; Sun et al. 2023; Nwogbaga and Camley 2024; Kaiyrbekov and Camley 2025). The information regulating these decisions is encoded by the noisy binding activity of diffusing signals at membrane bound receptors (Kashyap et al. 2024; Mugler et al. 2016; Camley 2018; Berg and Purcell 1977; Aquino et al. 2016; Fancher and Mugler 2017).

The ability to orient towards a source has been demonstrated across many model systems, including yeast (Henderson et al. 2019; Lakhani and Elston 2017; Ismael et al. 2016), Dictyostelium (Parent and Devreotes 1999) and neutrophils (Millius and Weiner 2009; Servant et al. 2000; Levchenko and Iglesias 2002). In the case of chemotaxis in neutrophils, numerous experimental studies (Millius and Weiner 2009; Weiner et al. 1999; Servant et al. 2000) have demonstrated that cells rapidly identify the directionality of an instantaneous chemoattractant source (administered by pipette). For example, neutrophils demonstrate asymmetric recruitment of membrane components within five seconds of exposure to a 10μM concentration of the chemoattractant FMLP presented by a micropipette at around two cell radii distance (Weiner 2002; Weiner et al. 1999). The timescale of such a response is well before a steady-state gradient is established (Lindsay et al. 2023).

A commonality across these systems is the role of membrane bound G protein-coupled receptors (GPCRs) which bind to signaling molecules (Boltz et al. 2022) and transmit information to downstream signaling cascades. These “sensors” therefore act as intermediaries between extracellular chemoattractants and downstream pathways which either inhibit or initiate necessary cellular responses.

Previous experiments utilized chemoattractant concentrations which saturated GPCRs. However, as the precision of experimental measurement has expanded, it is now clear that GPCRs can be activated at remarkably low levels of chemoattractant concentrations (Civciristov et al. 2018), including at the femtomolar ($10^{-15}$ M) and attomolar ($10^{-18}$ M) levels (Civciristov and Halls 2019; Calabrese and Giordano 2021). At such low concentration levels, receptor engagement and consequently cellular signaling decision making, are regulated by a small number of discrete binding events governed by extreme first passage time statistics. This work expands upon several recent studies that have employed extreme first passage time theory to understand decision making processes in biological systems (Linn et al. 2024; Morgan and Lindsay 2023; Lawley and Johnson 2023; Newby and Allard 2016).

**Fig. 1:**
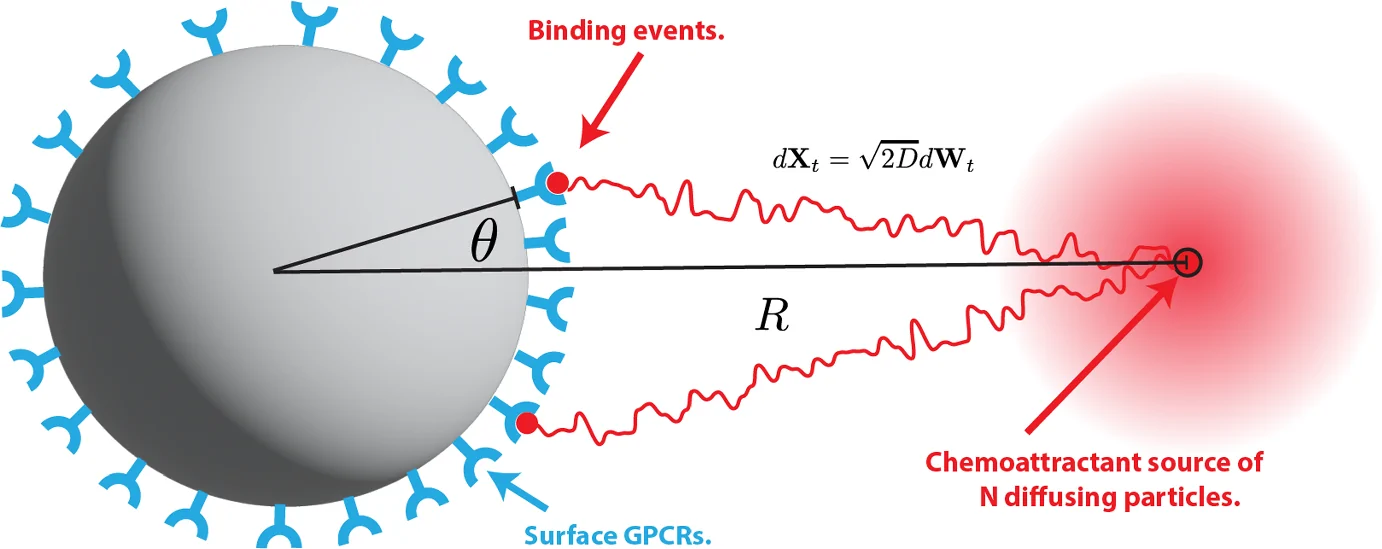
Schematic of source detection from the discrete arrival of signaling molecules to a circular cell. At t=0, a source located R>1 cell lengths away emits *N* diffusing molecules of diffusivity D>0. These signaling molecules diffuse freely before being absorbed at a surface receptor (GPCR). The extreme directional sensing problem is the following: given arrival times $\{T_{j,N}\}_{j=1}^k$ and corresponding polar locations $\{\theta _{j,N}\}_{j=1}^k$ of the first *k* impacts based on *N* initial walkers, what estimates can the cell form for the directionality of the source?

The aim of this paper is to give a mathematical analysis of the directional information that is encoded in signals associated with such low concentrations of chemoattractant. To this end, we consider an idealized non-dimensional scenario akin to the experiments of Weiner (2002); Weiner et al. (1999) where a circular cell (normalized to unit radius) is centered at the origin while a source of diffusing chemoattractant is placed at $\textbf{x}_0 = R[\cos \theta _0,\sin \theta _0]$ for R>1 (see Fig. 1). The quantity *R* represents the multiple of the cell radius at which the source is placed. Intuitively, we expect that detection at larger values of *R* is more challenging than source detection for values R≈1. A key element of our approach in the low chemoattractant limit is to consider the signaling molecules to be discrete. In this setting we assume at the initial time t=0 that *N* molecules are released from the source and undergo unbiased random motion before eventually reaching a receptor on the cell surface. As an example of typical values, a release of 1mL of chemoattractant at femtomolar to attomolar concentrations would represent a release of approximately $N\approx 10^3-10^6$ molecules. In our analysis we will assume a dense covering of GPCRs so that a continuum approximation is valid.

Each of the *N* molecules released at the source eventually reaches the cell surface and binds a receptor, producing two observable quantities: the time of the binding event and its location on the cell surface. Directional sensing is then an inverse problem: from the earliest binding events, what can the cell infer about the source? We will show that the two observables carry complementary information. The arrival times reflect the source distance, while the arrival locations reflect the source direction. We introduce the formal notation for the sets of arrival times and locations in Sect. 2, where the extreme-value statistics of the earliest arrivals are developed.

The most pertinent biological information on the source is its direction relative to the cell. It was noticed in Lindsay et al. (2023) that when recovering the location of a diffusive source from the time-dependent signal, the short time signal (0<t≪1) gave a strong estimate on the directionality of the source but yielded poor information on the distance. Heuristically, this is because molecules which are quickly absorbed at the cell surface have likely taken very direct or near-ballistic paths. This idea was further explored in Bernoff et al. (2023) which showed in a three dimensional setting that the directional information is contained primarily in a small fraction of the earliest signals received by the cell. Impacts that arrive after sufficiently long sojourns have largely lost information about their initial location and therefore dilute the directional information carried by earlier arrivals. This observation highlights a challenge in recovering source direction from equilibrium quantities (such as splitting probabilities), since such estimators necessarily average over signals of lower informational quality (Lawley et al. 2020; Dobramysl and Holcman 2018a, b). By using only the early fraction of the signal, it was shown in Bernoff et al. (2023) that a simple average of just a few impact locations can provide an accurate directional estimate. Here we build on these studies by deriving the joint statistics of early receptor binding times and impact locations in 2D.

We find that the information a cell can extract about the source separates into two channels: the times of early binding events, which encode the source distance *R*, and the locations of those events, which encode the source direction $\theta _0$. Section 3 asks how accurately the source distance can be inferred from arrival times alone, using maximum-likelihood estimation and the associated Fisher information both to quantify the best achievable accuracy and to compare candidate estimators. Sections 4–5 turn to the directional problem, deriving the distribution of arrival locations and analyzing estimators of source direction. In both cases we quantify the achievable accuracy as a function of the number *k* of early binding events.

The main mathematical result of this work is a derivation of the limiting distribution of the first *k* arrivals in this extreme statistics regime. Specifically, in Sect. 4, we find that the impact location of the $k^{\textrm{th}}$ arrival to a circular cell has the limiting behavior

1 $$\begin{aligned} \theta _{k,N} \sim \mathcal {N}(\theta _0,\sigma ^2_{k,N}), \qquad \sigma ^2_{k,N} = \frac{2D}{R}\Big (b_N + a_N \log k \Big ), \end{aligned}$$

as $N\rightarrow \infty$. Here $a_N$ and $b_N$ are quantities found in terms of the limiting properties of the survival probability for a single particle which we also obtain. For the specific case of an all absorbing circular cell of unit radius, the leading order limiting behavior is found to be

2 $$\begin{aligned} \theta _{k,N} \sim \mathcal {N}(\theta _0,\sigma ^2_{k,N}), \qquad \sigma ^2_{k,N} \sim \frac{(R-1)^2}{RW}\left( 1 + \frac{2\log k}{1+W}\right) , \qquad W = W_0\Big ( \frac{2N^2}{\pi R} \Big ), \end{aligned}$$

as $N\rightarrow \infty$. This behavior is based on the limiting forms $a_N\sim (R-1)^2/(DW(1+W))$ and $b_N\sim (R-1)^2/(2DW)$ as $N\rightarrow \infty$. Here $W_0(z)$ is the principal branch of the LambertW function (Corless et al. 1996), which has behavior $W_0(z) = \log z - \log \log z+ \cdots$ as $z\rightarrow \infty$. Hence $W_0\sim 2\log N$ as $N\rightarrow \infty$. The result reveals the limits on the accuracy of directional inference based on the first few early arrivals together with the dependence on key parameters, including source distance (*R*), number of early arrivals (*k*) and the strength of the initial signal (*N*). To our knowledge, this is the first derivation and analysis of the joint asymptotic distribution of early hitting times and impact locations for diffusive capture in two dimensions.

A surprising consequence of (2) is that the breadth of angular space sampled by the early arrivals is independent of the single particle diffusivity *D*. We will demonstrate that a larger diffusivity delivers the $k^{\textrm{th}}$ impact proportionally sooner by exactly the factor by which it broadens the angular distribution. Hence the parameter *D* sets only the overall timescale of capture, not the geometry of the early-impact distribution.

Using the detailed statistical properties of the early arrivals, we investigate the accuracy of several estimates that a cell could form to infer the source direction. We find that the impact times largely inform on the source distance while the impact locations encode the directionality. Our analysis shows that a cell can employ simple averaging over the first *k* arrivals to reduce the variance (error) in the accuracy.

Maximum likelihood estimation (MLE) can be used to form more accurate estimates of the source (Endres and Wingreen 2009). However, implementation of these estimators necessitates additional computational capabilities, such as the solution of nonlinear equations. Our analysis is able to quantify the accuracy in a range of estimators, from simple averages to relatively sophisticated MLEs. As an example of our theory, we analyze the simple average of the first *k* impacts given by ${\textbf {n}}^{\text {res}} = \frac{1}{k}\sum _{j=1}^k (\cos \theta _j,\sin \theta _j)$. We find that the error $\rho ^{\text {res}}_{k} = 1-{\textbf {n}}^{\text {res}}\cdot (\cos \theta _0,\sin \theta _0)$ associated with this estimate has mean and variance

3a $$\begin{aligned} \mathbb {E}[\rho _k^{\text {res}}]&\approx \frac{D}{R}\Big (b_N + a_N \big (\log k -1\big )\Big ), \end{aligned}$$

3b $$\begin{aligned} \text {Var}[\rho _k^{\text {res}}]&\approx \frac{2D^2}{R^2k}\Big ( \big (a_N \log k + b_N -a_N \big )^2 + a_N^2 \Big ). \end{aligned}$$

The error (3a) grows with *k* since the first impact has the lowest error while subsequent individual arrivals have logarithmically growing error. However, while the error increases in *k*, the variance in the error (3b) decreases. These opposing trends are a bias–variance trade-off. By considering the mean squared error $\mathbb {E}[(\rho _k^{\text {res}})^2]$, we show that this trade off results in an optimal $k = k^{*}$ which satisfies a transcendental equation. As $N\rightarrow \infty$, this critical number has asymptotic behavior $k^{*} \sim \log N$. For $k>k^{*}$, additional impacts produce a larger error in the resultant ${\textbf {n}}^{\text {res}}$. Hence an accurate estimate of the source direction can be formed from relatively few impacts *k*.

The remainder of the article is structured as follows. In Sect. 2 we describe preliminary results on the distributions of extreme arrival times, including those of individual arrivals and the joint distribution of *k* arrivals $\mathcal {T}_N$ and their differences. We emphasize that it is necessary to first describe the statistics of arrival times before arrival locations since the former depends on the latter. In Sect. 3 we introduce and analyze several estimates of the source distance using only the arrival times. These quantities primarily encode information on the distance of the source. In Sect. 4, we derive the distribution (1) of angular arrivals to a circular cell in the case of both Dirichlet and Robin boundary conditions. In Sect. 5, we use the result (1) to investigate the accuracy of several estimators of the source direction. Finally, in Sect. 6 we discuss future avenues of investigation that arise from this study.

## Statistics of extreme first passage times

In this section, we introduce key results on the distribution of the set of extreme hitting times which have been a topic of significant recent research in the literature (Linn et al. 2024; Linn and Lawley 2022; MacLaurin and Newby 2025; Tung and Lawley 2025). It is necessary to characterize the hitting times first before the hitting locations. This is due to the fact that particles arriving quickly to the cell have hitting distributions tightly focused on the source direction, while particles that undergo longer sojourns are distributed more evenly over the cell surface.

We denote the arrival times of the *N* molecules by $\{T_{j,N}\}_{j=1}^N$ and their arrival locations on the circular cell by $\{(\cos \theta _{j,N}, \sin \theta _{j,N})\}_{j=1}^N$, where the index j=1,…,N labels the molecules and *N* is the number released.

For an independent and identically distributed (i.i.d.) sequence of first passage times $\{\tau _1,\ldots ,\tau _N \}$ with survival probability $S(t):=\mathbb {P}(\tau _1>t)$, the first arrival is defined as

$$ T_{1,N}:= \min \{\tau _1,\ldots , \tau _N \}. $$

The i.i.d. property of the times means that

$$ \mathbb {P}(T_{1,N}>\tau ) = \mathbb {P}(\tau _1>\tau ) \times \cdots \times \mathbb {P}(\tau _N>\tau ) = [S(\tau )]^N. $$

For N≫1 and $0\le S(\tau )\le 1$, the mass becomes concentrated near t=0. Hence, describing extreme statistics requires detailed knowledge of the short time asymptotic behavior of *S*(*t*) as $t\rightarrow 0^{+}$. For a large class of diffusion problems in various dimensions, geometric settings and boundary conditions (Lawley 2020a; Linn and Lawley 2022) this behavior can be mapped onto the family of functions

4 $$\begin{aligned} 1-S(t) \sim At^p e^{-\frac{C}{t}}, \quad \text{ as } \quad t\rightarrow 0^{+}. \end{aligned}$$

Here *A*, *C*, *p* are constants with *A* and *C* being strictly positive. A simple example we will make extensive use of is diffusion on the half line with release at x=R and capture at x=0. The survival probability is $S(t) = 1 - \mathop \mathrm{{erfc}}(\frac{R}{\sqrt{4Dt}})$ and the limiting behavior as $t\rightarrow 0^{+}$ is given by

$$ 1 - S(t) \sim \sqrt{\frac{4Dt}{\pi R^2}} e^{\frac{-R^2}{4Dt}}, \qquad \text{ as } \qquad t\rightarrow 0^{+}. $$

More generally, the $k^{\textrm{th}}$ fastest arrival time at the cell is defined as

5a $$\begin{aligned} T_{k,N} := \min \big \{ \{\tau _1,\ldots , \tau _N\} \setminus \cup _{j=1}^{k-1} \{T_{j,N}\} \big \}, \qquad k \in \{1,\ldots ,N \}. \end{aligned}$$

Refined statistics on the distributions of the $k^{\textrm{th}}$ arrival $T_{k,N}$ have recently been determined in the limit as $N\rightarrow \infty$. A key result (Lawley 2020b, Thm. 4) utilized in the present work is the following convergence in distribution,

5b $$\begin{aligned} \left( \frac{T_{1,N}-b_N}{a_N},\ldots ,\frac{T_{k,N}-b_N}{a_N} \right) \rightarrow _\text {d}{\textbf {X}}^{(k)} := (X_1,\ldots , X_k)\in \mathbb {R}^k, \quad \text{ as } \quad N\rightarrow \infty . \end{aligned}$$

Here the joint probability density function of ${\textbf {X}}^{(k)}\in \mathbb {R}^k$ is

6 $$\begin{aligned} f_{{\textbf {X}}^{(k)}}(x_1,\ldots ,x_k) = \left\{ \begin{array}{rc} \exp (-{e^{x_k}}) \prod _{r=1}^k e^{x_r}, \quad & x_1\le \cdots \le x_k;\\ 0, \quad & \text{ otherwise. }\end{array}\right. \end{aligned}$$

The scale parameters $a_N$ and $b_N$ set the distribution of the early arrival times in the limiting law (). The constants $a_N$ and $b_N$ are defined as

7a $$\begin{aligned} b_N = \frac{C}{\log (AN)}, \quad a_N&=\frac{b_N}{ \alpha }, \qquad \alpha = \log (AN) ,&\text{ if } \quad p = 0;\end{aligned}$$

7b $$\begin{aligned} b_N = \frac{C}{pW},\quad a_N&= \frac{b_N}{\alpha }, \qquad \alpha = p(1+W),&\text{ if } \quad p \ne 0. \end{aligned}$$

We will show these quantities are defined in terms of the source distance and diffusivity so that estimating $a_N$ from the arrival times is therefore equivalent to estimating the source distance *R* for fixed *D*. The constant *W* is defined as

8 $$\begin{aligned} W = \left\{ \begin{array}{rl} W_0\big ( (C/p) (AN)^{1/p} \big ), & \quad p>0,\\ W_{-1}\big ( (C/p) (AN)^{1/p} \big ), & \quad p<0, \end{array}\right. \end{aligned}$$

where $W_0(z)$ and $W_{-1}(z)$ denote the principal and lower branches of the LambertW function respectively. The principal branch of the LambertW function has large argument asymptotic representation (Corless et al. 1996)

9 $$\begin{aligned} W_0(z) = \log z - \log \log z + \mathcal {O}\left( \frac{\log \log z }{\log z}\right) + \cdots \quad \text{ as } \quad z \rightarrow \infty . \end{aligned}$$

In the case p>0, we will often invoke the leading order behavior $W\sim \frac{1}{p}\log N$ as $N\rightarrow \infty$ to obtain expressions that are less accurate but offer more biological interpretability. To retain qualitative accuracy at moderate values of *N*, the full form described in (8) is retained.

For the individual arrival times, we have that the $k^{\textrm{th}}$ fastest arrival has the limiting behavior

10 $$\begin{aligned} \frac{T_{k,N}-b_N}{a_N} \rightarrow _\text {d}X_k, \quad \text{ as } \quad N\rightarrow \infty , \end{aligned}$$

where $X_k$ has the probability density function

11 $$\begin{aligned} f_{X_k}(x) = \frac{\exp (kx-e^x)}{(k-1)!}. \end{aligned}$$

**Fig. 2:**
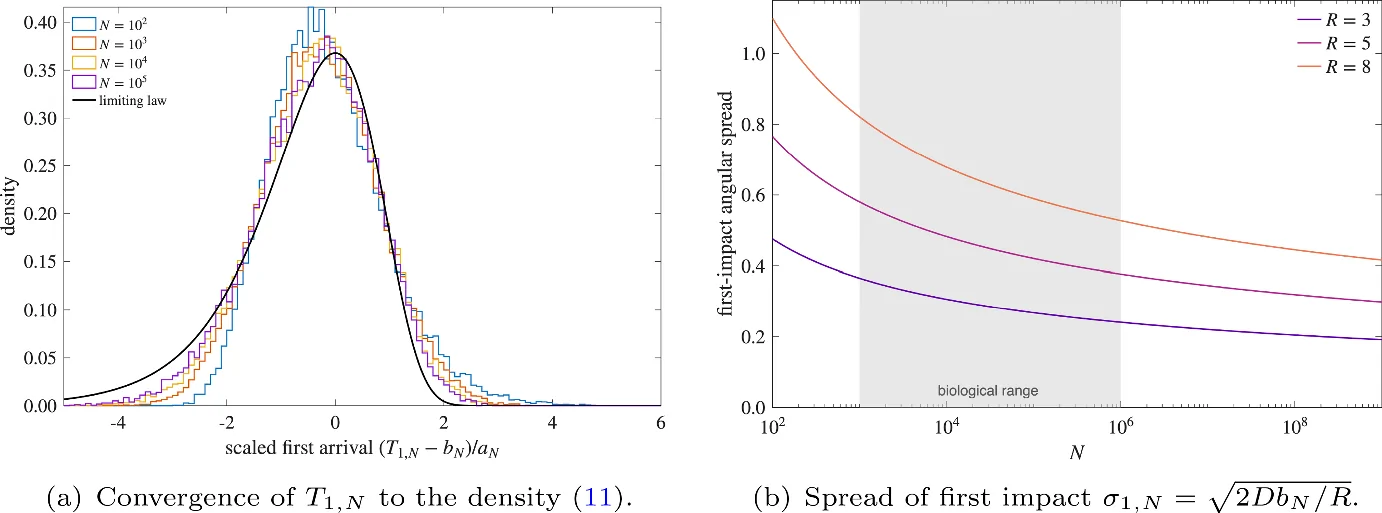
Convergence to the limiting distribution as $N\rightarrow \infty$. In Panel **a**, we show convergence of empirical distributions of $(T_{1,N}-b_N)/a_N$ to the limiting distribution $f_{X_1}(x) = \exp (x-\exp (x))$ for a range of *N*. In panel **b**, we plot the angular spread (1) of the first arrival $\sigma _{1,N} = \sqrt{2Db_N/R}$ to the cell. Over several decades in *N* we observe that the limiting distribution accurately captures the low concentration limit and that the angular spread varies modestly, hence providing a robust measure of directional sensing capabilities

In particular, we have that (Theorem 5 of Lawley 2020b)

12a $$\begin{aligned} \mathbb {E}[T_{k,N}]&= b_N + \psi (k)a_N + o(a_N),\end{aligned}$$

12b $$\begin{aligned} \text{ Var }(T_{k,N})&= \psi '(k) a_N^2 + o(a_N^2), \end{aligned}$$

where $\psi (k)= H_{k-1} - \gamma _e$ is the digamma function, $H_k = \sum _{r=1}^k\frac{1}{r}$ is the $k^{\textrm{th}}$ harmonic number, and $\gamma _e \approx 0.577$ is the Euler-Mascheroni constant. In Fig. 2a we show Monte-Carlo simulations that demonstrate the convergence of $T_{1,N}$ to the limiting distribution (11) for R=5 and D=1 in the case of the one-dimensional survival probability $S(t) = 1 - \mathop \mathrm{{erfc}}(\frac{R}{\sqrt{4Dt}})$. Over the biologically relevant range of *N* ($10^3-10^6$), we observe that the two distributions have broad agreement in shape. This is in agreement with related convergence studies performed in Lawley (2020b). In addition, we plot in Fig. 2b the quantity $\sigma _{1,N} = \sqrt{2Db_N/R}$ which characterizes the angular extent of the first impact. This quantity was stated in (1) and will be derived in Sect. 4. The key observation is that this quantity exhibits modest variations in value while the number of source particles *N* varies over several decades. Hence the biologically relevant range of values for *N* is well captured in the asymptotic limit $N\rightarrow \infty$.

The full record of arrival times $\{ T_{j,N} \}_{j=1}^k$ is a set of dependent random variables satisfying the joint distribution () in the limit as $N\rightarrow \infty$. This implies that a cell would need to store a sequence of times in order to process them to reconstruct information on the source. As a consequence, it is natural to ask whether simpler measurements of time can be used for inference. To this end, we derive (see Appendix 1) the distribution of the differences between arrival times. For $1\le k\ll N$, we have that the time differences are exponentially distributed, specifically,

13 $$\begin{aligned} T_{k+1,N}-T_{k,N} \sim _{\text {d}} Exp \left[ \frac{k}{a_N}\right] , \end{aligned}$$

with mean $a_N/k$ where $a_N$ is given in (). Hence, if we only know the interarrival times, then this value is related to the single parameter $a_N$ which shapes the limiting distribution for N≫1.

## Maximum likelihood estimates of source location from extreme time data

In this section, we describe a maximum likelihood estimation (MLE) process for determining the source parameters from the arrival statistics. The arrival time statistics depend on the source only through $a_N$ which itself depends on *R* and *D*. Therefore inferring source distance reduces to estimating the single parameter $a_N$. The MLE and its Fisher information that we derive in this section are able to characterize the best achievable accuracy and allow for a systematic comparison of the information content by different summaries of the data: the full arrival times versus the differences.

To set the stage for our MLE approach, consider a single cellular observation of *k* impact times ${\mathcal {T}}^{(k)} = (T_{1},T_{2},\ldots ,T_{k})$ based on *N* initial walkers. To simplify notation, we omit in this section the dependence of both ${\mathcal {T}}^{(k)}$ and $a= a_N$ on *N*. Suppose we have *n* independent and identically distributed realizations $\{\mathcal {T}^{(k)}_1,\ldots ,\mathcal {T}^{(k)}_n\}$ of this observable, corresponding to repeated releases from a source at the same distance.

Maximum likelihood estimation infers the unknown parameter *a* by maximizing the likelihood of the observed data. For the case of the full set of arrival times, the full likelihood is the product over the *n* independent observations,

14 $$\begin{aligned} \mathcal {L}(a | \{{\mathcal {T}_j}^{(k)}\}_{j=1}^n) = \prod _{j=1}^{n}f_{ \mathcal {T}^{(k)}}( {\mathcal {T}_j}^{(k)} | a) \end{aligned}$$

where $f_{ \mathcal {T}^{(k)}}$ is the joint density of the first *k* arrivals for each observation and given explicitly in (6) after rescaling.

The maximum likelihood estimate $\hat{a}$ is the maximizer of (14). This method is consistent: given a sufficient number *n* of observations, it converges in the sense that Vaart (1998):

15 $$\begin{aligned} \sqrt{n}\big (\hat{a}- a) \rightarrow _\text {d}\mathcal {N}(0, I^{-1}), \qquad I(a) = -\mathbb {E} \Big [ \frac{\partial ^2}{\partial a^2} \log \mathcal {L}\big (a | {\mathcal {T}}^{(k)}\big ) \Big | \ a \Big ] \end{aligned}$$

as $n\rightarrow \infty$. The quantity *I*(*a*) is the (per-observation) *Fisher information*, a simple quantity that determines the quality of the estimation with larger values corresponding to a more accurate estimate on the correct value. The total information from *n* independent observations is *nI*(*a*).

In the following two sections we build estimators from two different summaries of this data. In Sect. 3.1 we use only the interarrival differences $T_{i+1}-T_i$, which are mutually independent (Appendix 1) and yield a particularly simple estimator; in Sect. 3.2 we use the full arrival times through the joint likelihood (14). Comparing their Fisher information quantifies how much information about the source distance is discarded by retaining only the interarrival differences.

### Assuming the cell accesses the time differences

In the scenario we consider the situation where the time interval between arrivals is exponentially distributed as given in (13). Let $\{{\mathcal {T}}^{(k)}_{1},{\mathcal {T}}^{(k)}_{2},\ldots ,{\mathcal {T}}_n^{(k)}\}$ be a set of *n* i.i.d. observations for first *k* arrival times after the release of *N* molecules at t=0. Here ${\mathcal {T}}^{(k)}_{j} = (T_{1,j},\ldots ,T_{k,j})$ where we have assumed the *k* arrival times are sorted $(T_{i,j}<T_{i+1,j})$. The dependence of *N* is not explicitly recorded. Under the assumption that the *n* observations are independent and that, within each observation, the successive interarrival times are mutually independent with $T_{i+1,j} - T_{i,j} \sim _d \text {Exp}(i/a)$ (rate $\lambda _i = i/a$; see Appendix 1), the joint probability density (likelihood) of the complete set of interarrival times

$$ \mathcal {L}\big (a|\{ \mathcal {T}_j^{(k)} \}_{j=1}^n\big ) = \prod _{j=1}^{n} \prod _{i=1}^{k-1} \lambda _i e^{-\lambda _i (T_{i+1,j}-T_{i,j})}, \qquad \lambda _i = \frac{i}{a}. $$

For convenience, the subscript on the unknown parameter $a = a_N$ is suppressed. The negative log of the likelihood is more amenable to analysis and is given by

$$ \ell \big (a|\{ \mathcal {T}_j^{(k)} \}_{j=1}^n\big ) = - \log \Big [\mathcal {L}\big (a|\{ \mathcal {T}_j^{(k)} \}_{j=1}^n \big )\Big ] $$

With this definition, we calculate that

$$\begin{aligned} \ell \big (a|\{ \mathcal {T}_j^{(k)} \}_{j=1}^n\big )&= -\log \prod _{j=1}^{n} \prod _{i=1}^{k-1} \lambda _i e^{-\lambda _i (T_{i+1,j}-T_{i,j})}\\&= \sum _{j=1}^{n}\sum _{i=1}^{k-1} \Big [ \log a + \frac{i}{a} (T_{i+1,j}-T_{i,j}) - \log i\Big ]\\&= n(k-1)\log a + \frac{1}{a}\sum _{j=1}^{n} \sum _{i=1}^{k-1} i (T_{i+1,j}-T_{i,j}) - n \sum _{i=1}^{k-1} \log i. \end{aligned}$$

The first and second derivatives with respect to *a* yield the score function and the Fisher information respectively. These quantities are given by

16a $$\begin{aligned} \frac{\partial \ell }{\partial a}&= \frac{n(k-1)}{a} - \frac{1}{a^2}\sum _{j=1}^{n} \sum _{i=1}^{k-1} i (T_{i+1,j}-T_{i,j})\end{aligned}$$

16b $$\begin{aligned} \frac{\partial ^2 \ell }{\partial a^2}&= -\frac{n(k-1)}{a^2} + \frac{2}{a^3}\sum _{j=1}^{n} \sum _{i=1}^{k-1} i (T_{i+1,j}-T_{i,j}). \end{aligned}$$

The zeros of the score function yield the MLE estimate $\hat{a}$ given by

17 $$\begin{aligned} \hat{a}_n = \frac{1}{n(k-1)}\sum _{j=1}^{n}\sum _{i=1}^{k-1}i (T_{i+1,j}-T_{i,j}). \end{aligned}$$

The Fisher information of the full observation set is given by

18 $$\begin{aligned} \mathbb {E}\left[ \frac{\partial ^2 \ell }{\partial a^2} \right] = \frac{n(k-1)}{a^2}, \end{aligned}$$

so that the per-observation information from a single independent observation is

$$\begin{aligned} I_{\text {diff}}(a) = \frac{k-1}{a^{2}}. \end{aligned}$$

To obtain (18), we have calculated that

$$\begin{aligned} \mathbb {E}\left[ \sum _{j=1}^{n}\sum _{i=1}^{k-1} i(T_{i+1,j}-T_{i,j}) \right] = na(k-1), \end{aligned}$$

where from (12a), we have used that $\mathbb {E}[T_{k,j}]= \mathbb {E}[T_{1,j}]+ H_{k-1}a$. Hence, applying the result (15), we conclude that the estimate (17) satisfies

19 $$\begin{aligned} \sqrt{n}(\hat{a}_n - a) \rightarrow _\text {d}\mathcal {N}\Big (0, \frac{a^2}{k-1}\Big ), \quad \text{ as } \quad n \rightarrow \infty . \end{aligned}$$

**Fig. 3:**
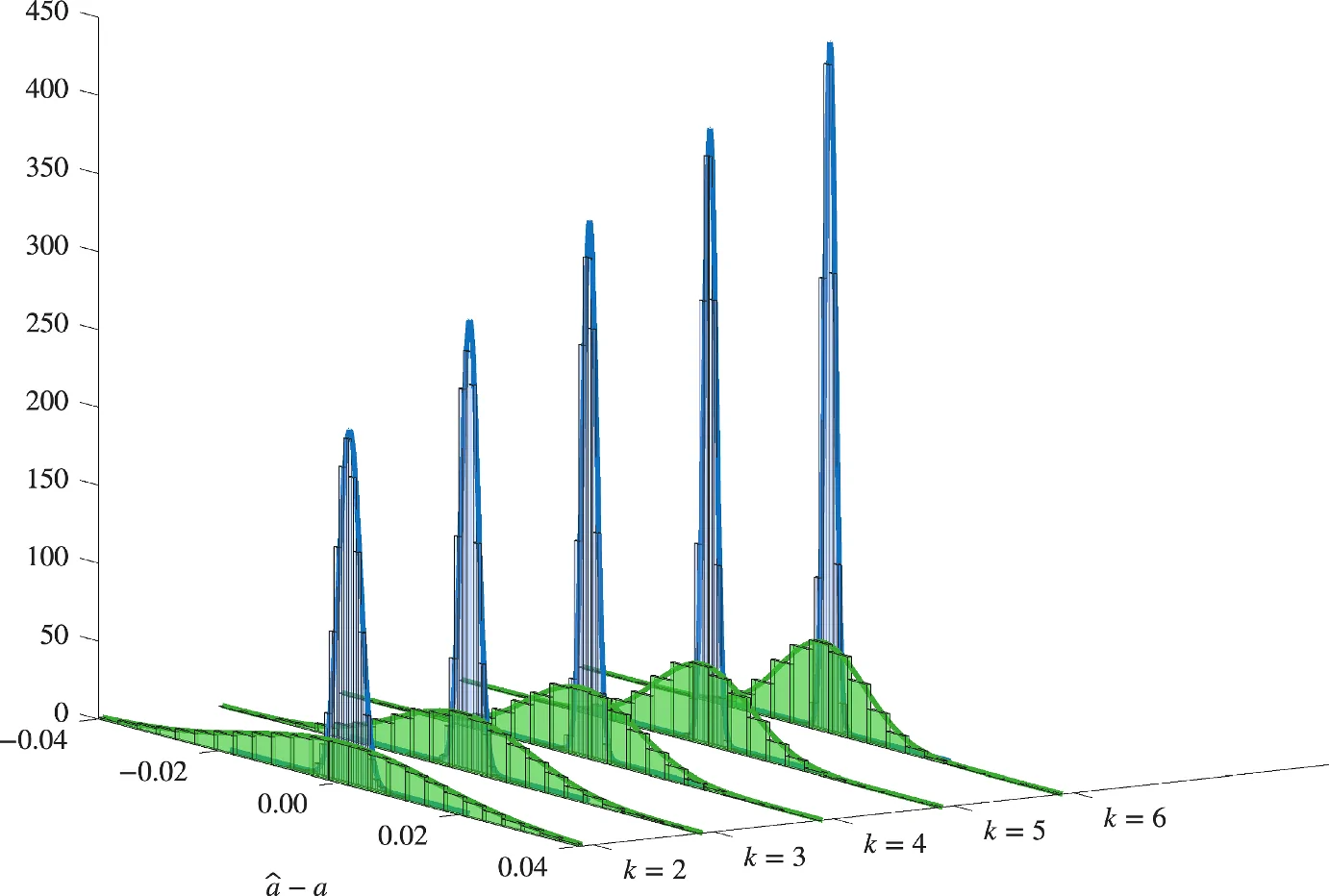
Recovery of the extreme distribution parameter for test case a=0.5 for $n = 10^3$. Distribution in errors $(\hat{a}-a)$ using the full times (blue) and the time differences (green). Each histogram is formed by $M= 10^4$ repetitions and for each we overlay the distribution $\mathcal {N}(0,1/\sqrt{nI(a)})$ where the Fisher Information *I*(*a*) is given in (29)

As a confirmation of the result (19), we simulated $n=10^3$ arrival events to create an estimate for $\hat{a}$. This process was repeated over $M=10^4$ trials to build a distribution for these estimates. In Fig. 3 we show excellent agreement between the theoretically predicted distribution (solid green) and that obtained by simulation (green histogram).

### Estimation using the full times

In this section, we describe a scenario where the cell can access the entire set of arrival times. The likelihood function of *n* independent arrival times $\{T_{1,j},T_{2,j},\ldots ,T_{k,j}\}$ for j=1,…,n, is given by

$$ \mathcal {L} (a | \{ \mathcal {T}_j^{(k)} \}_{j=1}^n) = \prod _{j=1}^{n}\frac{e^{-e^{x_{k,j}}}}{a^k}\prod _{i=1}^k e^{x_{i,j}}, \qquad x_{i,j} = \frac{T_{i,j} - \alpha a}{a}, $$

where α=p(1+W) is assumed constant. The negative log likelihood is given by

20 $$\begin{aligned} \ell = -\log \Bigg (\prod _{j=1}^{n}\frac{e^{-e^{x_{k,j}}}}{a^k} \prod _{i=1}^k e^{x_{i,j}} \Bigg )=nk \log a + \sum _{j=1}^{n}e^{\frac{T_{k,j}-\alpha a}{a}} - \sum _{j=1}^{n}\sum _{i=1}^k \frac{T_{i,j}-\alpha a}{a}. \end{aligned}$$

The first and second derivatives of ℓ are calculated to be

$$\begin{aligned} \frac{\partial \ell }{\partial a}&= \frac{nk}{a} -\frac{1}{a^2}\sum _{j=1}^{n}T_{k,j}e^{\frac{T_{k,j}-\alpha a}{a}} + \frac{1}{a^2}\sum _{j=1}^{n} \sum _{i=1}^k T_{i,j},\\ \frac{\partial ^2 \ell }{\partial a^2}&= -\frac{nk}{a^2} +\frac{1}{a^4}\sum _{j=1}^{n}(T_{k,j})^2e^{\frac{T_{k,j}-\alpha a}{a}} +\frac{2}{a^3}\sum _{j=1}^{n}T_{k,j}e^{\frac{T_{k,j}-\alpha a}{a}} -\frac{2}{a^3} \sum _{j=1}^{n}\sum _{i=1}^k T_{i,j}. \end{aligned}$$

The zeros of the score function yield the MLE estimate $\hat{a}$ which is given by

22 $$\begin{aligned} 0 = \hat{a} -\frac{1}{nk}\sum _{j=1}^n T_{k,j}e^{\frac{T_{k,j}-\alpha \hat{a}}{ \hat{a}}} +\frac{1}{nk}\sum _{j=1}^n\sum _{i=1}^kT_{i,j}. \end{aligned}$$

Introducing the change of variable *z* = $\frac{1}{ \hat{a}}$ reduces (22) to

23 $$\begin{aligned} e^\alpha - \frac{z}{nk}\sum _{j=1}^n T_{k,j}e^{zT_{k,j}} +\frac{z}{nk}e^\alpha \sum _{j=1}^n\sum _{i=1}^kT_{i,j} = 0. \end{aligned}$$

In this case, the Eq. (23) is nonlinear and we utilize a bisection method to access its roots $\hat{a}_n$. The Fisher information is

24 $$\begin{aligned} I(a)&= \mathbb {E}\left[ \frac{\partial ^2}{\partial a^2} \ell ({\mathcal { T}^{(k)}};a) \Big | a \right] \nonumber \\&= \mathbb {E}\left[ \sum _{j=1}^{n} \left( \Big (\frac{T_{k,j}^2}{a^4} + \frac{2T_{k,j}}{a^3}\Big ) e^{\frac{T_{k,j}-\alpha a}{a}}\right) \right] -\frac{2}{a^3} \mathbb {E}\Big [\sum _{j=1}^{n}\sum _{i=1}^k T_{i,j}\Big ] - \frac{nk}{a^2}\nonumber \\&= n \underbrace{\mathbb {E}\left[ \left( \Big (\frac{T_{k}^2}{a^4} + \frac{2T_{k}}{a^3}\Big ) e^{\frac{T_{k}-\alpha a}{a}}\right) \right] }_{I_1} - \frac{2n}{a^3} \underbrace{\mathbb {E}\Big [\sum _{i=1}^k T_{i}\Big ]}_{I_2} - \frac{nk}{a^2}. \end{aligned}$$

We now proceed to calculate the expectations $I_1$ and $I_2$ separately.

***Calculation of*** $$I_1$$:

The probability density function of $T_{k}$, given by (11), is

$$ \mathbb {P}(T_{k} = t) = \frac{e^{k(\frac{t - \alpha a}{a}) - e^\frac{t - \alpha a}{a}}}{a(k - 1)!}. $$

Hence, by defining the auxiliary functions *h*(*t*) and *g*(*t*) as

$$ h(t) = \frac{1}{a(k-1)!} \left( \frac{t^2}{a^4} + \frac{2t}{a^3}\right) , \qquad g(t) = (k+1)(t-\alpha a) - a e^{(a^{-1}(t-\alpha a))}, $$

we can express the integral $I_1$ as

$$ I_1 = \mathbb {E}\left[ \left( \Big (\frac{T_{k}^2}{a^4} + \frac{2T_{k}}{a^3}\Big ) e^{\frac{T_{k}-\alpha a}{a}}\right) \right] = \int \limits _{t=0}^{\infty } h(t) e^{a^{-1}g(t)}\text {d}t. $$

Applying Laplace’s method for a≪1, we approximate this integral as

$$\begin{aligned} I_1&\approx \sqrt{\frac{2\pi a}{|g''(t_c)|} }h(t_c) \exp [a^{-1}g(t_c)] \\&= a \sqrt{\frac{2\pi }{k+1}}h(t_c)\exp \Big [\big (k+1\big )\big (\log (k+1)-1\big )\Big ] \qquad \text{ for } \qquad a\ll 1. \end{aligned}$$

Here we have calculated

$$ t_c = a [\alpha + \log (k+1)], \qquad g({t_c}) = a(k+1) [\log (k+1)-1], \qquad |g''(t_c)| = \frac{k + 1}{a}. $$

After some rearrangement and simplification, we obtain that

25 $$\begin{aligned} I_1 \approx \sqrt{\frac{2\pi }{k+1}}\left( \frac{t_c^2}{a^4} + \frac{2 t_c}{a^3} \right) \frac{1}{(k-1)!} \left( \frac{k+1}{e}\right) ^{k+1}. \end{aligned}$$

This expression can be further simplified using Stirling’s approximation

$$ (k+1)!\approx \sqrt{2\pi (k+1)}\left( \frac{k+1}{e}\right) ^{k+1}, $$

which yields

26 $$\begin{aligned} I_1 = \Big ( \big (\log (k+1)+ 1 +\alpha \big )^2 -1\Big ) \frac{k}{a^2}. \end{aligned}$$

***Calculation of*** $$I_2$$:

Recalling from () that b=αa and from (12a) that $\mathbb {E}[T_{k}] \approx a\big (\alpha + \psi (k)\big )$, we calculate

$$\begin{aligned} I_2 = \mathbb {E}\left[ \sum _{i=1}^{k} T_{i} \right]&= k a \alpha + a\Big [ \psi (1) + \psi (2) + \cdots + \psi (k)\Big ]\\&= ka (\alpha - \gamma _e) + a\left[ \sum _{i=1}^{k-1} H_{i} \right] = ka(\alpha - \gamma _e) + a(kH_{k-1} -k+1), \end{aligned}$$

where we have used $\sum _{i=1}^{k-1}H_i = kH_{k-1} - (k-1).$ Combining $I_1$ and $I_2$ in (24) together with simplification, we obtain the Fisher information for the total observation of full times

27a $$\begin{aligned} \mathbb {E}\left[ \frac{\partial ^2 \ell }{\partial a^2} \right] = \frac{nk}{a^2}\Big ( \big (\log (k+1)+ 1 +\alpha \big )^2 - 2 \big ( H_{k-1} -\gamma _e + \alpha \big ) -\frac{2}{k}\Big ). \end{aligned}$$

If we consider an approximation for large *k*, we can apply $H_{k} \approx \log k +\gamma _e + \mathcal {O}(k^{-1})$ for k≫1 and obtain the following compact representation

27b $$\begin{aligned} \mathbb {E}\left[ \frac{\partial ^2\ell }{\partial a^2} \right] \approx \frac{nk}{a^2}\Big ( \big (\log k+\alpha \big )^2 +1 \Big ) . \end{aligned}$$

The per-observation Fisher information is then given by

28 $$\begin{aligned} I_{\text {full}} \approx \frac{k}{a^2}\Big ( \big (\log k+\alpha \big )^2 +1 \Big ). \end{aligned}$$

Hence we have derived two consistent estimators $\hat{a}$ based on either the difference in times (17) or the full arrival times (23). The approximate convergence behavior for these estimates as $n\rightarrow \infty$ is

29 $$\begin{aligned} \sqrt{n}(\hat{a}_n - a) \rightarrow _\text {d}\mathcal {N}(0,I^{-1}), \qquad I(a) = \frac{1}{a^2} \left\{ \begin{array}{lc} k-1, & \text{ time } \text{ differences; } \\ k\, (\log k + \alpha )^2 + k, & \text{ full } \text{ times, } \end{array} \right. \end{aligned}$$

where *I*(*a*) is the per-observation Fisher information.

Before proceeding to numerical validation of the main result (29), we make some comments on its biological significance. Clearly $I_{\text {full}}>I_{\text {diff}}$ for any k≥1 and hence knowledge of the full times yields more information on the source position. However, exploiting this fact requires storing these times and solving the nonlinear system (23). Conversely, a cell can utilize a simpler procedure of a weighted average of the inter-arrival times (17). While it exhibits higher variance (lower accuracy), this estimate requires substantially less computational work to obtain.

As a confirmation of the estimates (29), we take the fixed value of a=1/2 and generate $n = 10^3$ samples (from (6)) of the first *k* arrival times for k=2,3,4,5,6. From these observations, we create estimates $\hat{a}$ using both the MLE estimate based on the full times and the time differences. To explore the entire distribution predicted by (29) we then repeat this process $M=10^4$ times and plot the results in Fig. 3 and observe that our result (29) accurately predicts the distribution of errors. In addition, these results confirm that the full time method generates estimates with much lower error and that the error is further reduced as *k* increases.

**Fig. 4:**
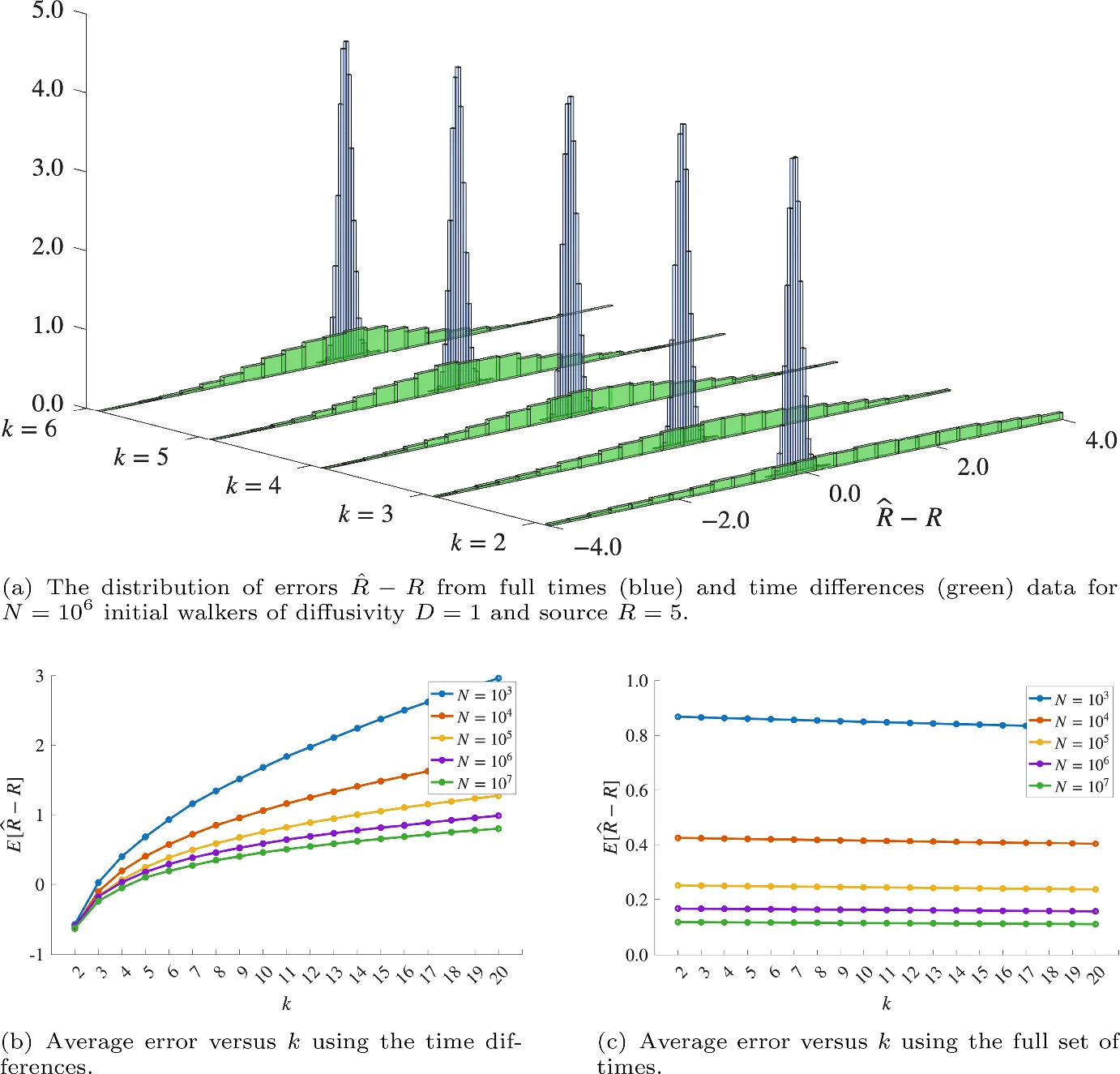
Accuracy in the prediction (30) of source distance using the full arrival times and the differences in arrival times in one-dimension. Panel **a**: The distribution of errors for source detection when R=5 and $N = 10^6$. The estimate is vastly more accurate using the full times (blue) than the time differences (green). Distributions formed from $M=10^4$ independent estimates. In panels **b**–**c** we plot the average error against *k* for initial walker numbers ranging from $N = 10^3-10^7$

### Finite *N*

The previous analysis and its demonstration were conducted purely on the limiting distribution as the number of walkers $N\rightarrow \infty$. To consider the problem for a finite number of walkers, we consider an idealized one-dimensional problem, where *N* walkers with diffusivity *D* originate at the point x=R and are absorbed at x=0. The survival probability for this situation is given by $S(t) = 1 - \mathop \mathrm{{erfc}}(\frac{R}{\sqrt{4Dt}})$. The limiting behavior as $t\rightarrow 0^{+}$ is given by

$$ 1 - S(t) \sim \sqrt{\frac{4Dt}{\pi R^2}} e^{\frac{-R^2}{4Dt}}, \qquad \text{ as } \qquad t\rightarrow 0^{+}. $$

By comparing with (4), we identify the constants

$$ A = \sqrt{\frac{4D}{\pi R^2}}, \qquad p = \frac{1}{2}, \qquad C = \frac{R^2}{4D}. $$

Taking these values and applying them to the result (), we obtain that

$$ a_N = \frac{R^2}{DW(1+W)}, \qquad W= W_0\left( \frac{2N^2}{\pi }\right) , $$

where $W_0(z)$ is the principal branch of the LambertW function. We additionally notice that $\alpha = \frac{1}{2}(1+ W)$ is independent of *R* and that $b_N= \alpha \, a_N$. Hence, given the first *k* arrival times, we can form an estimate $\hat{a}$, either using the full times or their differences, to arrive at an estimate

30 $$\begin{aligned} \hat{R} = [DW(1+W)\hat{a}]^{\frac{1}{2}}. \end{aligned}$$

In Fig. 4 we display results on the accuracy of the estimate $\hat{R}$ for a range of *k* and initial walker numbers *N* with the true value R=5. Across all examples, we see that exploiting the full arrival times yields a far more accurate estimate of the source distance *R*. The observation that the full-time distribution is far narrower than the difference-based one is clear from the ratio $I_{\text {full}}/I_{\text {diff}} \approx (\log k + \alpha )^2 \approx 10^2$ for the values in Fig. 4. In Fig. 4c we observe a very slight dependence on *k* as the error is dominated by *N* when the MLE estimator is so much more efficient. The algorithms we have introduced and analyzed in the previous section, are based on the limiting distribution of the arrival times in the limit as $N\rightarrow \infty$. In Fig. 4b–c we observe the convergence of the error for a sequence of initial walker number *N* and again notice that the full arrival time estimate performs markedly better than using only the differences.

## Estimation of source direction from extreme time data in 2D

In this section, we move on to the problem of estimating the source direction from the information contained in the first *k* arrival impacts. The arrival locations are time dependent since quick arrivals are more focused around the source direction whereas longer sojourns arrive more uniformly on the cell surface.

### Statistics of arrival locations for a circular cell

In order to determine the impact location distribution, we consider a circular cell of unit radius and a source placed at $\textbf{x}_0 = (R,0)$ for R>1. The placement of the source at an angle $\theta _0$ relative to the horizontal is a simple rotation in the counter-clockwise direction. In polar coordinates $\textbf{x}= r(\cos \theta ,\sin \theta )$, the quantity $u(r,\theta ,t)r\textrm{d}r\textrm{d}\theta$ is the probability of a particle being in $(r,r+\textrm{d}r) \times (\theta ,\theta + \textrm{d}\theta )$ at time *t*. Then u(r,θ,t) solves the diffusion equation

31a $$\begin{aligned} \frac{\partial u}{\partial t} = D\Big [\frac{\partial ^2 u}{\partial r^2} + \frac{1}{r} \frac{\partial u}{\partial r} + \frac{1}{r^2} \frac{\partial ^2 u}{\partial \theta ^2}\Big ], \quad r\in (1,\infty ), \quad \theta \in (-\pi ,\pi ) \quad t>0;\end{aligned}$$

31b $$\begin{aligned} u = \frac{1}{r}\delta (r-R)\delta (\theta ), \quad r\in (1,\infty ), \quad \theta \in (-\pi ,\pi )\quad t=0;\end{aligned}$$

31c $$\begin{aligned} u(r,\theta +2\pi ,t) = u(r,\theta ,t), \quad r\in (1,\infty ), \quad \theta \in (-\pi ,\pi ), \quad t \ge 0;\end{aligned}$$

31d $$\begin{aligned} {\partial _{\theta }u(r,\theta +2\pi ,t) = \partial _{\theta }u(r,\theta ,t), \quad r\in (1,\infty ), \quad \theta \in (-\pi ,\pi ), \quad t \ge 0.} \end{aligned}$$

The choice of boundary conditions on the surface of the cell can be influenced by many factors including the distribution and affinities of the receptors. For the purposes of the present study, we consider a continuum of receptors evenly distributed on the cellular surface. Under these conditions, and assuming $N_{\text {rec}}$ receptors of non-dimensional common radius ${\displaystyle \varepsilon }_{\text {rec}}$, the following Robin boundary condition has recently (Lindsay et al. 2023) been derived

31e $$\begin{aligned} D\frac{\partial u}{\partial r} = \kappa u, \quad r = 1; \qquad D\kappa ^{-1} = -\frac{2}{N_{\text {rec}}} \log \Big (\frac{N_{\text {rec}}{\displaystyle \varepsilon }_{\text {rec}}}{4}\Big ). \end{aligned}$$

When the number of receptors is large, $(N_{\text {rec}}\approx 10^5)$, each with radius $10^{-8}-10^{-10}m$ (Odoemelam et al. 2020), the value of κ is large, resulting in an effectively fully absorbing boundary. In the limiting $\kappa \rightarrow \infty$ case, the Dirichlet boundary condition u=0 can be applied on r=1. Further analysis on the homogenization of porous membranes is described in Brennan et al. (2026); Lindsay et al. (2015).

The treatment of receptors as a uniform distribution rather than considering their discrete positions (e.g. Lindsay et al. (2023); Lawley et al. (2020)) affords many simplifications. It is frequently observed that receptors can have non-uniform surface distributions that are modulated by the curvature of the membrane (Alonso et al. 2025). Hence, the analysis of the uniform case presented here serves as a baseline for future studies on the role of geometry in chemotaxis.

With the assumption of the uniform boundary condition (31e), we are able to determine the short time asymptotics of the solution to (). The two key quantities that describe the hitting times and hitting locations, are the survival probability *S*(*t*) and surface flux ${\mathcal {J}}(\theta ,t)$, respectively. These quantities are defined from the solution of () by

32a $$\begin{aligned} S(t)&= \int \limits _{r=1}^{\infty } \int \limits _{\theta =-\pi }^{\pi }u(r,\theta ,t) \, r\, \text {d}r\, \text {d}\theta \end{aligned}$$

32b $$\begin{aligned} {\mathcal {J}}(\theta ,t)&=D\partial _ru(r=1,\theta ,t). \end{aligned}$$

To derive the short time asymptotic forms of () as t→0, we apply the method of moments (Lindsay et al. 2023). The first step is to apply the Laplace transform $\hat{u}(r,\theta ;s)=\int \limits _{t=0}^{\infty } u(r,\theta ,t)e^{-st} \text {d}t$ to Eq. (). We follow a standard process of writing the solution in terms of the cosine-Bessel series (Stepien et al. 2020) where we enforce continuity and a jump condition at r=R, require the solution to be finite as $r\rightarrow \infty$ and also enforce the boundary condition (31e). The Laplace transform of the flux is found to be

33 $$\begin{aligned} \hat{{\mathcal {J}}}(\theta ;s) = \frac{1}{2\pi } \chi _0(q) + \frac{1}{\pi }\sum _{n=1}^{\infty } \chi _n(q) \cos n\theta , \qquad \chi _n(q) = \frac{\kappa \big (K_n(q R)/K_n'(q)\big ) }{\kappa \big (K_n(q)/K'_n(q)\big ) - D q}. \end{aligned}$$

where $q = \sqrt{s/D}$. Our goal is to approximate the solution of (33) for $q\rightarrow \infty$ which corresponds to the limit $t\rightarrow 0^{+}$ in the time domain. We define the moments $\widehat{M}_n(s) = \int \limits _{-\pi }^{\pi }\hat{{\mathcal {J}}}(\theta ;s)\theta ^n \text {d}\theta$ and apply $\cos n\theta \approx 1 - \frac{1}{2}n^2\theta ^2 + \frac{1}{24} n^4 \theta ^4$ in (33) to obtain

34 $$\begin{aligned} \chi _n(q) = \int \limits _{-\pi }^{\pi } \hat{{\mathcal {J}}}(\theta ;s) \cos n \theta \, \text {d}\theta \approx \widehat{M}_0(s) - \frac{n^2}{2} \widehat{M}_2(s) + \frac{n^4}{24} \widehat{M}_4(s). \end{aligned}$$

The large argument asymptotics (Abramowitz and Stegun 1964, §9.7.2) of $K_n(z)$ for $z\rightarrow \infty$ yields the behavior

$$\begin{aligned} \frac{K_n(q R)}{K_n'(q)} \sim&\frac{e^{-q(R-1)}}{R^{\frac{1}{2}}} \left[ -1 + \frac{ (4n^2+3) (R-1) +4}{8Rq} \ + \right. \\&\left. \frac{(-16n^4 + 8n^2 -33)(R-1)^2 + (32n^2-72)(R-1) + (64n^2 -48)}{128 R^2q^{2}} \right] . \end{aligned}$$

Notice that the term $K_n(q)/K'_n(q)$ arises from substituting R=1 into this expression. We insert this approximation into the expression for $\chi _n$ given in (33) and then apply to (34). Gathering terms at the relevant orders of *n*, we obtain the following expressions for the moments

$$ \widehat{M}_0 \sim \frac{\kappa e^{-q(R-1)}}{R^{\frac{1}{2}}(\kappa + D q)}, \quad \widehat{M}_2 \sim \frac{\kappa (R-1) e^{-q(R-1)}}{R^{\frac{3}{2}}q(\kappa + D q)}, \quad \widehat{M}_4 \sim \frac{3\kappa (R-1)^2 e^{-q(R-1)}}{R^{\frac{5}{2}}q^2(\kappa + D q)}, $$

in the limit as $q \rightarrow \infty$. Inverting these Laplace transforms explicitly as $q\rightarrow \infty$ ($t\rightarrow 0^{+}$), we identify the following limits for the variance and kurtosis of the flux

$$ \text {Var}[{\mathcal {J}}(\theta ,t)]:= \frac{M_2}{M_0} \sim \frac{2 Dt}{R}, \qquad \text {Kur}[{\mathcal {J}}(\theta ,t)]:= \frac{M_4 M_0}{[M_2]^2} \sim 3, \qquad t \rightarrow 0^{+}. $$

The kurtosis is the normalized fourth moment $\text {Kur}[\mathcal {J}] = M_4M_0/M_2^2$, equal to 3 for a Gaussian; its convergence to 3 as $t\rightarrow 0^{+}$ confirms the limiting flux is Gaussian. Hence, in the limit as $t\rightarrow 0^{+}$, we have derived from () the short time behavior

35a $$\begin{aligned} {\mathcal {J}}(\theta ,t) \sim \frac{M_0(t)}{\sigma \sqrt{2\pi }} e^{-\frac{\theta ^2}{2\sigma ^2}}, \qquad \sigma ^2 \sim \frac{2Dt}{R}, \end{aligned}$$

as $t\rightarrow 0^{+}$. Here the total arrival rate $M_0(t)$ at the cell surface is given by

35b $$\begin{aligned} M_0(t) \sim \left\{ \begin{array}{ll} \frac{\kappa (R-1)}{\sqrt{\pi R Dt}} e^{-\frac{(R-1)^2}{4Dt}}\left[ \frac{1}{2\kappa t + (R-1) }\right] , & \qquad \kappa \in (0,\infty );\\ \frac{(R-1)}{\sqrt{4\pi R Dt^3}} e^{-\frac{(R-1)^2}{4Dt}} & \qquad \kappa = \infty , \end{array} \right. \end{aligned}$$

as t→0. The survival probability satisfies $S'(t) = -\int \limits _{\theta =-\pi }^{\pi } {\mathcal {J}}(\theta ,t) \text {d}\theta \sim -M_0(t)$. From this relationship, we obtain that

35c $$\begin{aligned} S(t) \sim 1 - \left\{ \begin{array}{ll} \frac{4\kappa }{ \sqrt{\pi R} } \frac{\sqrt{D} }{ (R-1)^2 } t^{\frac{3}{2}} e^{-\frac{(R-1)^2}{4Dt}}, & \qquad \kappa \in (0,\infty );\\ \frac{ \sqrt{4D} }{ (R-1)\sqrt{\pi R} } t^{\frac{1}{2}} e^{-\frac{(R-1)^2}{4Dt}}, & \qquad \kappa = \infty . \end{array} \right. \end{aligned}$$

Hence, comparing between (35c) and (4), the constants from the short time survival probability $S(t) \sim 1- At^p e^{-\frac{C}{t}}$ that characterize the distribution of arrival times are

36 $$\begin{aligned} \begin{array}{llll} p = \frac{3}{2}, & \quad A = \frac{4\kappa }{ \sqrt{\pi R} } \frac{\sqrt{D} }{ (R-1)^2 }, & \quad C = \frac{(R-1)^2}{4D}, & \qquad \kappa \in (0,\infty );\\ p = \frac{1}{2}, & \quad A = \frac{ \sqrt{4D} }{ (R-1)\sqrt{\pi R} }, & \quad C = \frac{ (R-1)^2 }{ 4D }, & \qquad \kappa = \infty . \end{array} \end{aligned}$$

These variables now define the scalars $a_N$ and $b_N$ that characterize the distribution of arrival times through the definitions ().

### The angular distribution of impacts

Here we analyze the $k^{\textrm{th}}$ arrival location characterized by the impact time $T_{k,N}$ and determine the distribution of the corresponding angular location $\theta _{k,N}$. As discussed previously, the distribution of the angular location depends on the time of impact. Hence, we have that

$$ \mathbb {P}[\theta _{k,N} = \Theta ] = \int \limits _{t=0}^\infty \mathbb {P}[\theta _{k,N} = \Theta | T_{k,N}=t] \mathbb {P}[T_{k,N} = t] \text {d}t. $$

The limiting distribution for $\mathbb {P}[T_{k,N}= t]$ is given in (11) and takes the form

$$ \mathbb {P}[T_{k,N}= t] = \frac{1}{a_N(k-1)!}\exp [\varphi (t)], \qquad \varphi (t) = \frac{k(t-b_N)}{a_N} - \exp \left[ \frac{t-b_N}{a_N}\right] . $$

The distribution of angular arrivals now satisfies

$$\begin{aligned} \mathbb {P}[\theta _{k,N} = \Theta ] = \frac{1}{a_N(k-1)!} \int \limits _{t=0}^\infty \mathbb {P}[\theta _{k,N} = \Theta | T_{k,N}=t] \exp [\varphi (t)]\text {d}t. \end{aligned}$$

In the limit as $N\rightarrow \infty$, which corresponds to $a_N\rightarrow 0$, we apply Laplace’s method to simplify this integral. The value of the integral is dominated by the sharp peak of the function φ(t) around the peak (mode) $t_c =b_N + a_N \log k$. In the vicinity of $t_c$, the function has a local quadratic shape with $\varphi ''(t_c)= -k/a_N^2$. This allows for a standard application of Laplace’s method. From the distribution of fluxes along the surface of the cell derived in (35a), we conclude that

37 $$\begin{aligned} \mathbb {P}[\theta _{k,N} = \Theta \ | \ T_{k,N}= t_c ] = \frac{1}{\sigma \sqrt{2\pi }} e^{-\frac{\theta ^2}{2\sigma ^2}}, \qquad \sigma ^2 \sim \frac{2Dt_c}{R}. \end{aligned}$$

**Fig. 5:**
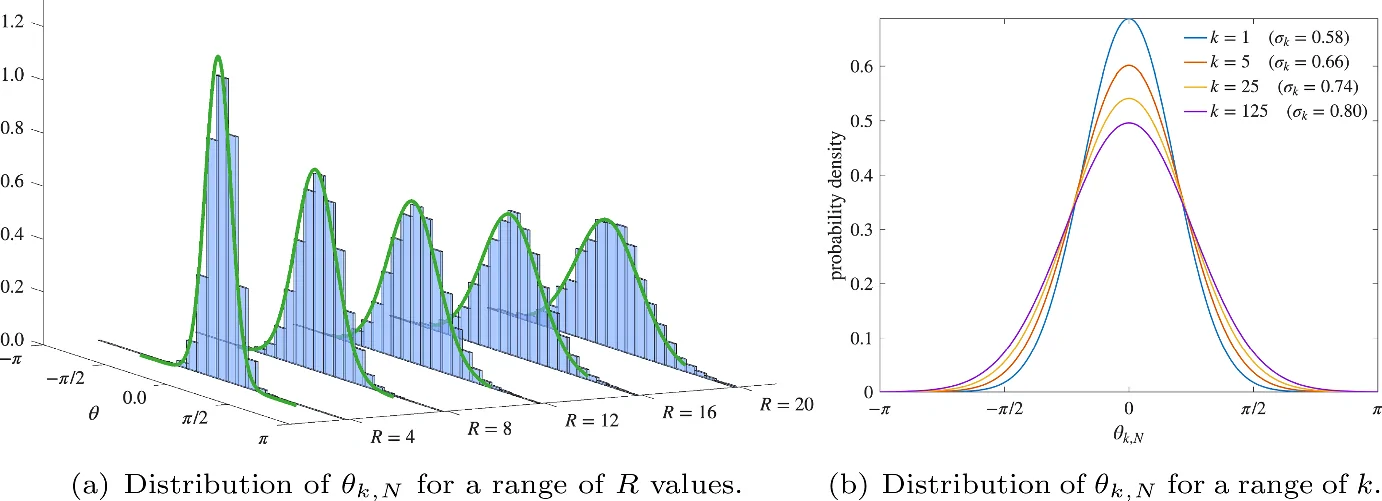
The distribution of impact angles $\theta _{k,N}$. Panel a shows results for k=10, D=1, $\theta _0 = 0$ and $N= 10^6$ for various *R*. The histograms are based on $M=10^4$ impacts sampled directly from the case of Dirichlet boundary condition ($\kappa = \infty$). The solid curve is the predicted limiting distribution (38) with good agreement observed. In panel b we show the limiting impact distribution (38) with fixed parameters R=5, D=1, $N=10^3$ and over a range of *k* values. The distribution of arrival angles broadens as *k* increases

Finally, we conclude that the impact locations have a Gaussian distribution given by

38 $$\begin{aligned} \theta _{k,N} \sim \mathcal {N}\big (\theta _0, \sigma _{k,N}^2\big ), \qquad \sigma _{k,N}^2 = \frac{2D}{R}\Big (b_N + a_N \log k \Big ), \end{aligned}$$

in the limit as $N\rightarrow \infty$. From Eq. (38) we observe that since $a_N>0$, particles arriving earlier to the cell have impact locations more tightly distributed (lower variance) around the source direction. Consequently, the first particle to arrive at the cell has the most directional information, followed by the second, and then followed by the third, etc. As a confirmation of this, we plot in Fig. 5a a comparison between particle simulations of the arrival distribution and the result (38). In Fig. 5b we plot the limiting distribution of the $k^{\textrm{th}}$ impact (38) for fixed *R* and a range of *k* values.

The variance in the impact location (38) is independent of the particle diffusivity *D*. This reflects a cancellation of two competing effects: the angular spread of the flux conditioned on arrival at time *t* grows as $2Dt_c/R$ while the characteristic arrival time of the $k^{\textrm{th}}$ molecule, $t_c = b_N + a_N \log k$, scales as 1/*D*. A larger diffusivity therefore delivers the $k^{\textrm{th}}$ impact proportionally sooner by exactly the factor by which it broadens the angular distribution. The result is that the $k^{\textrm{th}}$ arrival always samples the same range of angular space hence *D* sets the timescale of capture but not the geometry of the early-impact distribution.

## Recovery of source direction from extreme arrivals

In this section, we use the main result (38) to explore the accuracy of several estimators on the direction of the source.

### Assuming the cell accesses and averages the full angle values

Let us consider a situation where the cell uses the first *k* angles $\{\theta _{j,N}\}_{j=1}^k$ from *N* initial walkers to form an estimate of the source direction. Without loss of generality, we set the source to have direction $\theta _0=0$. A first quantity to consider is the average angle $\Theta ^{\text {avg}}_k$ inferred by these impacts. It has the following definition:

39 $$\begin{aligned} \Theta ^{\text {avg}}_k = \frac{1}{k}\sum _{j=1}^k \theta _{j,N}, \qquad [\text {Average angle}]. \end{aligned}$$

Applying the distribution of each $\theta _{j,N}$ for j=1,…,k given in (38), we obtain that

40 $$\begin{aligned} \Theta ^{\text {avg}}_k \sim \mathcal {N}\left( 0, \frac{2D}{Rk^2}\Big (b_Nk + a_N\log k! \Big )\right) \approx \mathcal {N}\left( 0, \frac{2D}{Rk}\Big (b_N -a_N + a_N\log k\Big ) \right) , \end{aligned}$$

where on the last step we have applied the approximation $\log k! \approx k\log k - k$. We notice that by increasing the number *k* of points averaged, the variance decreases like $k^{-1}\log k$. This indicates that although the variance associated with individual impacts increases with *k*, the averaged variance is overall decreasing.

### Estimates based on the difference in angles

Here we consider an estimate based on the average of the differences in the angle between impacts. Specifically, we derive the distribution of

41 $$\begin{aligned} \Theta _k^{\text {diff}} = \frac{1}{k}\sum _{j=1}^{k-1} \big [ \theta _{j+1,N} - \theta _{j,N} \big ] \end{aligned}$$

From (38), we have that $\theta _{k,N}\sim \mathcal {N}(0,\sigma _{k,N}^2)$, and hence we observe that

42 $$\begin{aligned} \theta _{j+1,N} - \theta _{j,N} \sim \mathcal {N}\left( 0, \frac{2D}{R}\Big (b_N + a_N\log \big (j+1\big )\Big ) + \frac{2D}{R}\Big (b_N + a_N \log \big (j\big )\Big )\right) . \end{aligned}$$

By summing the first k-1 differences in angle we get the following simplification

$$\begin{aligned} \sum _{j=1}^{k-1} \big [\theta _{j+1,N} - \theta _{j,N} \big ]&= (\theta _{2,N} - \theta _{1,N}) + (\theta _{3,N} - \theta _{2,N}) + \cdots + (\theta _{k,N} - \theta _{k-1,N})\\&= \theta _{k,N} - \theta _{1,N}. \end{aligned}$$

The average of the differences then has the distribution

43 $$\begin{aligned} \Theta _k^{\text {diff}} = \frac{1}{k}\sum _{j=1}^{k-1} \big [ \theta _{j+1,N} - \theta _{j,N} \big ] = \frac{1}{k} (\theta _{k,N}-\theta _{1,N})\sim \mathcal {N}\left( 0, \frac{2D}{Rk^2}\big (2b_N + a_N\log k\big )\right) . \end{aligned}$$

**Fig. 6:**
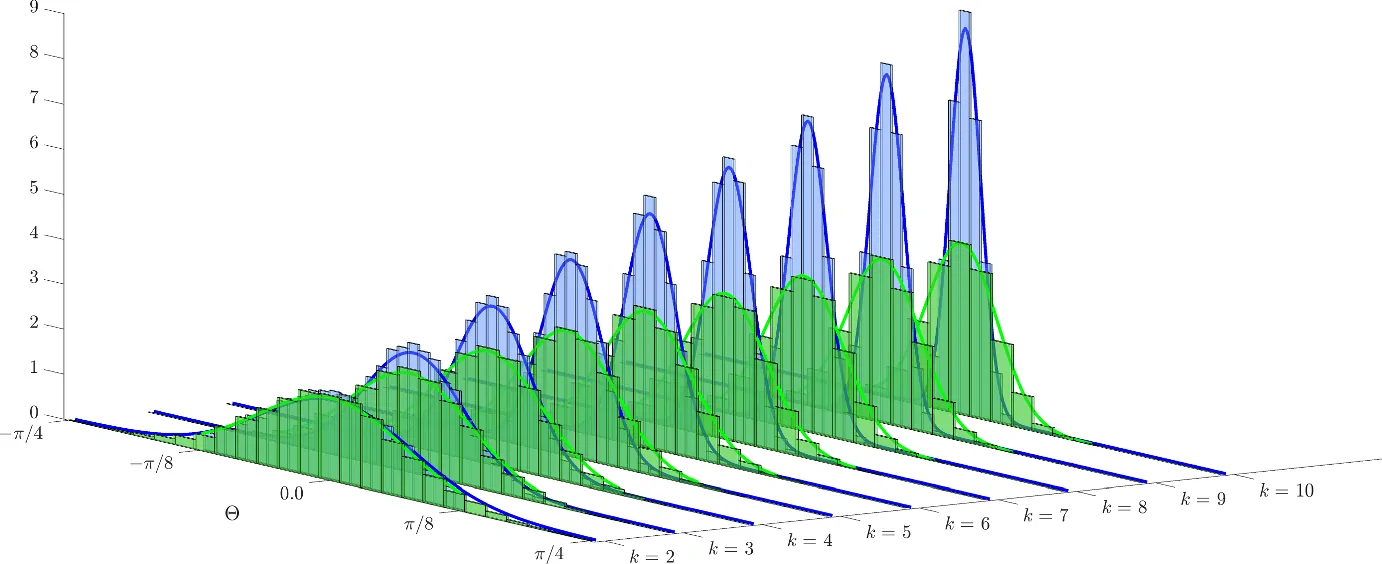
Distribution of angular estimates in predictions using the average angles $\Theta _k^{\text {avg}}$ (green histograms) defined in (40) and the differences estimate $\Theta _k^{\text {diff}}$ (blue histograms) defined in (43)

In Fig. 6 we show confirmation of the expressions (40) for the average $\Theta _k^{\text {avg}}$ and (43) for the average difference $\Theta _k^{\text {diff}}$. We generated $M=10^4$ samples of the $k^{\textrm{th}}$ arrival angle $\theta _{k,N}$ to a circular cell based on $N=10^6$ initial walkers. These were sampled exactly from the closed form flux expression (33) by applying a numerical inverse Laplace transform (see Cherry et al. 2025). We observe that for k=1, both estimates provide similar accuracy on the source direction. However, as *k* increases the difference estimate (43) improves because fewer values are averaged.

### The statistics of the resultant vector

In this example, we analyze the resultant formed by averaging over a vector of each arrival. Specifically, let

$$ {\textbf {n}}^{\text {res}} \equiv \frac{1}{k} \sum _{j=1}^k {\textbf {n}}_j, \qquad {\textbf {n}}_j = \big (\cos \theta _j, \sin \theta _j\big ), \qquad j = 1,\ldots ,k, $$

be the equally weighted average of the first *k* impacts at the cell. When utilizing a polar coordinate system where the source has location $\textbf{x}_0 = R(1,0)$, the true source is in the direction ${\textbf {e}}_1 = (1,0)$. Hence, the angular difference between the vector ${\textbf {n}}^{\text {res}}$ and the source is estimated by

44 $$\begin{aligned} \Theta ^{\text {res}} = \left<{ {\textbf {n}}^{\text {res}},{\textbf {e}}_1 }\right> = \frac{1}{k} \sum _{j=1}^k \cos \theta _j. \end{aligned}$$

In the limit as $N\rightarrow \infty$, we assume the angular distribution is tightly distributed around the region $|\theta |\ll 1$. Expanding for $|\theta _j|\ll 1$, we have that

45 $$\begin{aligned} \Theta ^{\text {res}} = \frac{1}{k} \sum _{j=1}^{k} \cos \theta _j \approx 1 - \frac{1}{2k}\sum _{j=1}^k \theta _j^2. \end{aligned}$$

The expression (45), which represents a combination of squared Gaussian random variables, is known to follow the *generalized chi-squared* distribution. In the simpler scenario in which $\theta _j$ are i.i.d., the random variable follows the chi-squared distribution.

Hence the error $\rho _k^{\text {res}}$ in the resultant vector formed by *k* arrivals is described by the generalized chi-squared variable (Das 2025)

46 $$\begin{aligned} \rho _k^{\text {res}} = \frac{1}{2k}\sum _{j=1}^k \theta _j^2 = \frac{D}{Rk} \sum _{j=1}^{k} \big (b_N+a_N\log (j)\big )Z_j^2, \end{aligned}$$

where $Z_j\sim \mathcal {N}(0,1)$. The expected value and variance of this random variable satisfy

47a $$\begin{aligned} \mathbb {E}[\rho _k^{\text {res}}]&= \frac{D}{Rk} \sum _{j=1}^{k} \big (b_N+a_N\log j\big ) \approx \frac{D}{R}\Big (b_N + a_N \big (\log k -1\big )\Big ), \end{aligned}$$

47b $$\begin{aligned} \text {Var}[\rho _k^{\text {res}}]&= \frac{2D^2}{R^2k^2} \sum _{j=1}^{k} \big (b_N+a_N\log j\big )^2 \approx \frac{2D^2}{R^2k}\Big ( (a_N \log k + b_N -a_N )^2 + a_N^2 \Big ). \end{aligned}$$

In arriving at (), we have applied the approximations

$$ \sum _{j=1}^{k} \log j \approx k\log k - k, \qquad \sum _{j=1}^{k} [\log j]^2 \approx k\,\big (\log ^2 k - 2\log k + 2\big ), \qquad k\gg 1. $$

**Fig. 7:**
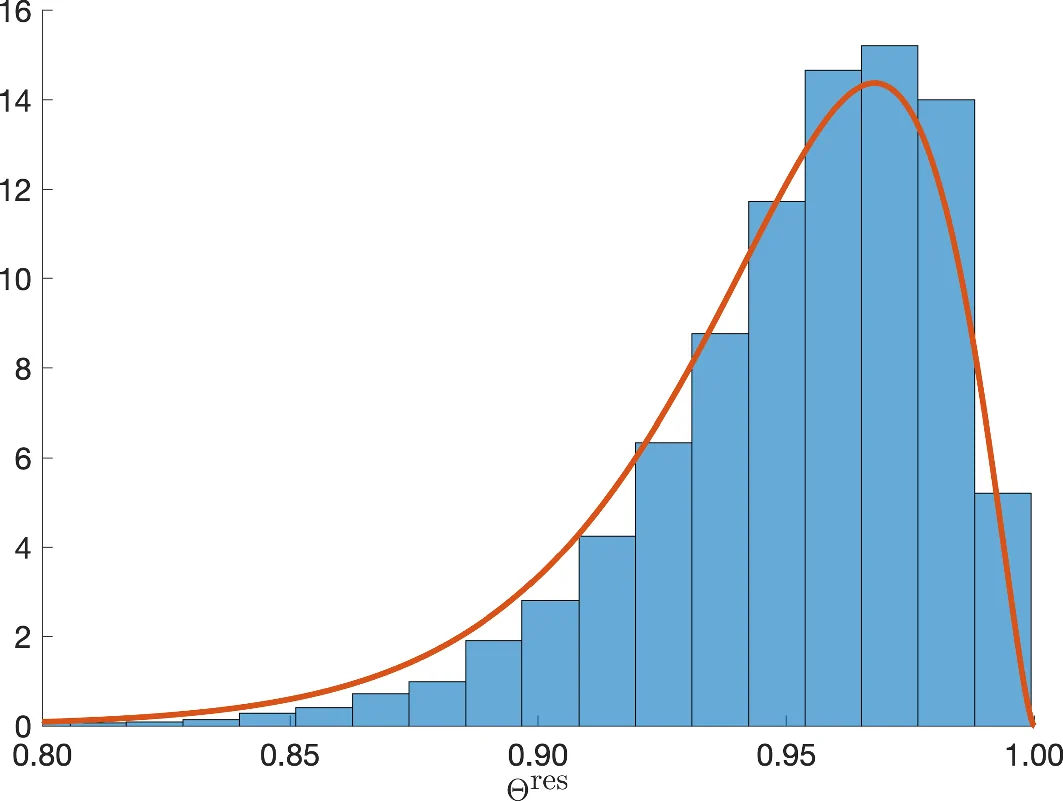
The distribution of the resultant vector based on the average of the first *k* arrivals for k=5 and $N= 10^6$ initial walkers at a distance of R=4. The histogram shows estimates $\Theta ^{\text {res}} = {{\textbf {n}}}^{\text {res}} \cdot {\textbf {e}}_1$, defined in (44) for $M=10^4$ realizations. The generalized chi-squared distribution (solid) defined in (45) gives a close approximation to the estimate

In Fig. 7, we plot results for the estimate $\Theta ^{\text {res}}$ for R=4 and k=5. We see that the distribution of the resultant is closely approximated by the generalized chi-squared distribution (46). This good agreement validates the predicted mean and variance of the estimator.

**Fig. 8:**
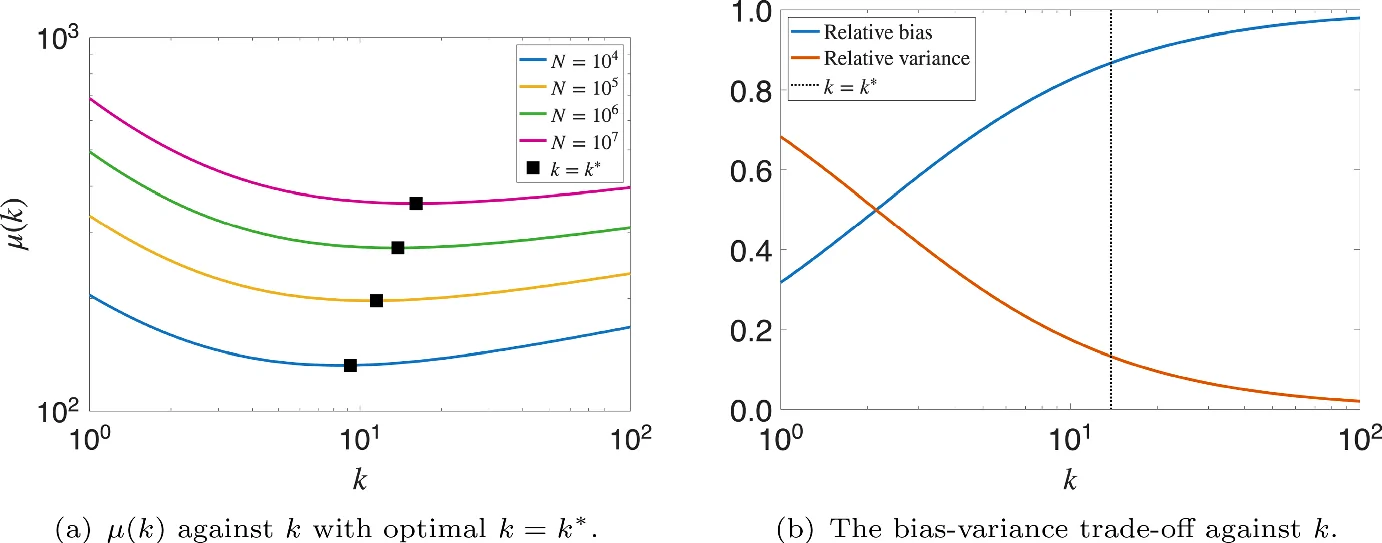
Bias-variance trade-off in the mean squared error (MSE) (48) as a function of impacts *k* in the case $\alpha = \log N$. In panel **a** we plot μ(k) against *k* for several values of *N*. We observe the MSE is minimized for $k^{*} \approx \log N$. In panel **b**, we fix $N= 10^6$ and show the relative importance of bias and variance (51) in the MSE as *k* increases. For $k<k^{*}$, the MSE is decreasing as *k* increases. Conversely, for $k>k^{*}$, the MSE increases with the number of impacts

***Bias-variance trade-off for finite*** *k*

The observation that the error (47a) increases gradually with *k*, while the variance (47b) decreases suggests a classic bias-variance trade-off where a small number of arrivals can combine in a simple vector average to give an accurate estimate of the source direction. To investigate this further, we calculate the mean squared error (MSE), to be

48 $$\begin{aligned} \mathbb {E}\big [(\rho _k^{\text {res}})^2\big ]&= \mathbb {E}\big [\rho _k^{\text {res}}\big ]^2 + \text {Var}\big [\rho _k^{\text {res}}\big ]\nonumber \\&= \frac{D^2}{R^2}\left[ \Big (b_N + a_N \big (\log k -1\big )\Big )^2 + \frac{2}{k} \Big ( \big (a_N(\log k-1) + b_N\big )^2 + a_N^2 \Big )\right] \nonumber \\&= \frac{D^2a_N^2}{R^2} \mu (k), \qquad \Big [\, \mu := \Big (\alpha + \log k - 1\Big )^2 \Big (1+ \frac{2}{k}\Big ) + \frac{2}{k} \, \Big ], \end{aligned}$$

where we have used $b_N/a_N = \alpha = p(1+W)$ from (). The critical points $k^{*}$ of the mean squared error satisfy $\mu '(k^{*})=0$. After algebra, this condition reduces to

49 $$\begin{aligned} k^{*} = \alpha + \log k^{*} - 3 + \frac{1}{\alpha + \log k^{*} -1}. \end{aligned}$$

For $k^{*}$ fixed and N≫1, we have W≫1 and $\log k^{*} \ll W$. In Appendix 2 we use these properties to find that the solution of (49) to have behavior

50 $$\begin{aligned} k^*=\log N +(1-p)\log \log N +[p+\log (AC^p) -3]+o(1)\quad \text {as}\quad N\rightarrow \infty . \end{aligned}$$

Remarkably, we find that in the regime N≫1, the optimal number of receptor events is $k^{*}\approx \log N$, independent of geometric factors which provide $\mathcal {O}(1)$ corrections. In Appendix 2 we explore the dependence of $k^{*}$ on these lower order corrections for some particular choices of boundary conditions.

In Fig. 8 we display results for the case $\alpha \approx \log N$. In Fig. 8a we show the minimization of the MSE (48) with *k* for several values of N≫1. The critical values $k^{*} \approx \log N$ accurately capture the threshold beyond which further impacts increase the MSE. In Fig. 8b, we plot the relative bias and relative variance for fixed $N=10^6$. These quantities are defined as

51 $$\begin{aligned} \text {Rel. bias}: \ \frac{\mathbb {E}\big [\rho _k^{\text {res}}\big ]^2}{\mathbb {E}\big [\rho _k^{\text {res}}\big ]^2+\text {Var}\big [\rho _k^{\text {res}}\big ]}, \quad \text {Rel. variance:} \ \frac{\text {Var}\big [\rho _k^{\text {res}}\big ]}{\mathbb {E}\big [\rho _k^{\text {res}}\big ]^2 + \text {Var}\big [\rho _k^{\text {res}}\big ]}. \end{aligned}$$

As seen in Fig. 8b, the bias in the estimate increases with *k* while the variance decreases with *k*. The vertical line $k= k^{*} \approx \log N$ is the critical value beyond which more impacts deteriorate the estimate. In Fig. 8a the optimization landscape is seen to be relatively shallow indicating that for even k≈10, the MSE is very close to that of the optimal choice $k^{*}$ over several decades in *N*.

## Discussion

In this work, we investigated the limits of single-cell directional sensing in the ultra-low chemoattractant regime, where receptor binding is discrete and cellular decisions occur long before steady state. A central mathematical contribution of this study is the derivation and validation of explicit asymptotic distributions for both the hitting times and hitting locations of diffusing signaling molecules at the cell surface. By coupling extreme first-passage time statistics with short-time angular localization, we obtained a joint statistical description of early receptor binding events that is valid in the biologically relevant pre-equilibrium regime.

The biological significance of this mathematical framework is that it provides a quantitative framework for comparing the directional information available to the cell under different hypothesized sensing mechanisms. Rather than assuming equilibrium gradients or time-averaged receptor occupancy, our results make it possible to directly analyze how much information is encoded in specific features of early binding data, such as first-arrival times, interarrival intervals, binding locations, or combinations thereof. This enables a systematic comparison of inference strategies, including statistically optimal estimators and simpler biologically plausible estimators, on equal footing. As an example, we found that the interarrival times conveyed much less information to the cell than the full arrival times, but exploiting the full arrival record requires nonlinear processing of the arrival times.

Our analysis demonstrates that directional information is strongly front-loaded in early arrivals: molecules that bind soon after release are preferentially localized around the true source direction, while later arrivals lose directional bias. Additionally, source distance is primarily encoded in the arrival times while the source direction is encoded in arrival locations. As a result, accurate directional inference can be achieved from a small number of early binding events without the need for long-time averaging or high receptor occupancy. This provides a plausible mechanism that explains the experimentally observed rapid symmetry breaking and polarization responses that occur on timescales much shorter than those required to establish steady-state concentration profiles.

A central simplification of our analysis is the treatment of the cell surface as a continuum of receptors, encoded in the homogenized Robin condition (31e) with effective permeability κ. This description is justified when receptors are numerous and densely and uniformly distributed, for which $\kappa \gg 1$ and the surface is effectively absorbing (Lindsay et al. 2023). In this regime the fine-grained arrangement of individual receptors is averaged out, and the arrival statistics depend only on the source geometry and the bulk diffusion. Receptor polarization is characterized by a non-uniform surface density, possibly modulated by membrane curvature (Alonso et al. 2025) or as an active element of the downstream chemotactic response (Nwogbaga and Camley 2024). Capturing these effects requires moving beyond the homogenized boundary condition, for instance through discrete-trap or narrow-escape treatments (Lawley et al. 2020) or a matched-asymptotic analysis that resolves individual receptors while retaining the short-time structure in the survival probability. This is an important direction for extending the present results to more biologically realistic receptor configurations.

Several other extensions of this framework remain open for further analysis. In particular, source detection in environments containing multiple chemoattractant sources presents new challenges, as early arrivals may reflect competing extreme events whose statistics overlap in time and space (Kashyap et al. 2024). In congested environments, it is known that a community of cells can collaborate to better decipher environmental cues (Fancher and Mugler 2017; Camley et al. 2016; Camley 2018; Copos et al. 2025). Likewise, crowded or heterogeneous environments may modify first-passage statistics through obstruction, intermittency, or anomalous transport. Extending the joint hitting-time and hitting-location analysis to these settings would enable a quantitative assessment of how robust early-time directional sensing remains under realistic environmental complexity. Finally, an interesting avenue for future research will be to extend these results to more general cell shapes to further inform on the observed roles that aberrations to radial symmetry can play in cellular signaling processes (Chakraborty et al. 2025; Bernoff and Lindsay 2025; Kaiyrbekov and Camley 2025; Copos et al. 2025; Nwogbaga and Camley 2024).

## Data Availability

This manuscript has no associated experimental data. The code used to generate the figures and the numerical results are available from the corresponding author upon request.

## Extreme interarrival times are exponential

As stated in Eq. () (see also Lawley 2020b, Thm. 4), the times $\{T_{1,N},\ldots ,T_{k,N}\}$ obey the following convergence in distribution,

52 $$\begin{aligned} \bigg (\frac{T_{1,N}-b_N}{a_N},\dots ,\frac{T_{k,N}-b_N}{a_N}\bigg ) \rightarrow _\text {d}(X_1,\dots ,X_k)\quad \text {as }N\rightarrow \infty , \end{aligned}$$

where the probability density function $f_{{\textbf {X}}^{(k)}}$ of ${\textbf {X}}^{(k)} = (X_1,\dots ,X_k)$ is given in (6). The continuous mapping theorem ensures that continuous functions preserve convergence in distribution (Billingsley 2013). Hence, applying the following continuous function $g:\mathbb {R}^k\rightarrow \mathbb {R}^{k-1}$,

$$\begin{aligned} g(x_1,\dots ,x_k) =(x_2-x_1,\dots ,x_k-x_{k-1}), \end{aligned}$$

to (52) yields the following convergence in distribution,

$$\begin{aligned} \bigg (\frac{T_{2,N}-T_{1,N}}{a_N},\dots ,\frac{T_{k,N}-T_{k-1,N}}{a_N}\bigg ) \rightarrow _\text {d}(X_2-X_1,\dots ,X_k-X_{k-1})\,\,\text {as }N\!\rightarrow \!\infty . \end{aligned}$$

It thus remains to determine the joint distribution of $(X_2-X_1,\dots ,X_k-X_{k-1})$. For k=2, we have that for z≥0,

$$\begin{aligned} \mathbb {P}(X_2-X_1>z) =\mathbb {P}(X_2>X_1+z)&=\int \limits _{-\infty }^\infty \text {d}x_2\,e^{x_2}\exp (-e^{x_2})\int \limits _{-\infty }^{x_2-z}\text {d}x_1\,e^{x_1}\\&=e^{-z}\int \limits _{-\infty }^\infty \text {d}x_2\,e^{2x_2}\exp (-e^{x_2})=e^{-z}. \end{aligned}$$

Hence, $X_2-X_1$ is an exponential random variable with unit mean. For k=3, we have that for $z_1\ge 0$, $z_2\ge 0$,

$$\begin{aligned}&\mathbb {P}(X_3\!-\!X_2\!>\!z_2,\, X_2\!-\!X_1\!>\!z_1) \!=\!\mathbb {P}(X_3\!>\!X_2\!+\!z_2,\,\\&\quad X_2\!>\!X_1\!+\!z_1)\\&\quad =\int \limits _{-\infty }^\infty \text {d}x_3\,e^{x_3}\exp (-e^{x_3})\int \limits _{-\infty }^{x_3-z_2}\text {d}x_2\,e^{x_2}\int \limits _{-\infty }^{x_2-z_1}\text {d}x_1\,e^{x_1}\\&\quad =e^{-z_1}\int \limits _{-\infty }^\infty \text {d}x_3\,e^{x_3}\exp (-e^{x_3})\int \limits _{-\infty }^{x_3-z_2}\text {d}x_2\,e^{2x_2}\\&\quad =e^{-z_1-2z_2}\int \limits _{-\infty }^\infty \text {d}x_3\,e^{3x_3}\exp (-e^{x_3})/2 =e^{-z_1-2z_2}. \end{aligned}$$

Hence, $2(X_3-X_2)$ and $(X_2-X_1)$ are i.i.d. exponential random variables with unit means. For a general k≥2, we have that for $z_1\ge 0,\dots ,z_{k-1}\ge 0$,

$$\begin{aligned}&\mathbb {P}(X_k\!-\!X_{k\!-\!1}\!>\!z_{k\!-\!1},\dots ,X_2\!-\!X_1\!>\!z_1) \!=\!\mathbb {P}(X_k\!>\!X_{k\!-\!1}\!+\!z_{k\!-\!1},\dots ,X_2\!>\!X_1\!+\!z_1)\\&=\int \limits _{-\infty }^\infty \text {d}x_k\, e^{x_k}\exp (-e^{x_k})\int \limits _{-\infty }^{x_k-z_{k-1}}\text {d}x_{k-1}\,e^{x_{k-1}} \int \limits _{-\infty }^{x_{k-1}-z_{k-2}}\text {d}x_{k-2}\,e^{x_{k-2}}\cdots \int \limits _{-\infty }^{x_2-z_{1}}\text {d}x_{1}\,e^{x_{1}}\\&=e^{-z_1-2z_2-3z_3-\cdots -(k-1)z_{k-1}}\int \limits _{-\infty }^\infty \text {d}x_k\, e^{kx_k}\exp (-e^{x_k})/((k-1)!)\\&=e^{-z_1}e^{-2z_2}e^{-3z_3}\cdots e^{-(k-1)z_{k-1}}. \end{aligned}$$

Here, we have used that

$$\begin{aligned}&\int \limits _{-\infty }^{x_k-z_{k-1}}\text {d}x_{k-1}\,e^{x_{k-1}}\int \limits _{-\infty }^{x_{k-1}-z_{k-2}}\text {d}x_{k-2}\,e^{x_{k-2}}\cdots \int \limits _{-\infty }^{x_2-z_{1}}\text {d}x_{1}\,e^{x_{1}}\\&\quad =e^{-z_1-2z_2-3z_3-\cdots -(k-1)z_{k-1}}e^{(k-1)x_k}/((k-1)!), \end{aligned}$$

which can be established by induction. We have also used that the change of variables $u=e^x$ yields

$$\begin{aligned} \int \limits _{-\infty }^\infty \text {d}x_k\, e^{kx_k}\exp (-e^{x_k}) =\int \limits _0^\infty \text {d}u\,u^{k-1}e^{-u} =\Gamma (k)=(k-1)!. \end{aligned}$$

We therefore conclude that

$$\begin{aligned}&\big \{i(X_{i+1}-X_i)\big \}_{i=1}^{k-1} \\&\quad =\Big \{X_2-X_1,2(X_3-X_2),3(X_4-X_3),\dots ,(k-1)(X_k-X_{k-1})\Big \} \end{aligned}$$

are i.i.d. exponential random variables with unit means.

### $X_1$ and $X_2-X_1$ are dependent

Suppose we want to sample $(X_1,\dots ,X_k)$ from the pdf in (6). As a first attempt, one might first sample $X_1\sim \text {Gumbel}(0,1)$ and then let $X_2=X_1+E$ where *E* is an exponential random variable independent of $X_1$. However, this sampling method assumes that $X_1$ and $X_2-X_1$ are independent. However, this is false. In particular, while all the *inter*arrival times are independent, the first arrival time and the first interarrival time are *not* independent. This is intuitive; if the first particle takes a long time to hit the target (i.e. $X_1$ is large), then it seems likely that the first interarrival time will be small (i.e. $X_2-X_1$ is small). To see that $X_1$ and $X_2-X_1$ are dependent, we compute

$$\begin{aligned} \mathbb {E}[X_1(X_2-X_1)]&=\int \int x_1(x_2-x_1)f(x_1,x_2)\,\text {d}x_1\,\text {d}x_2 =-1-\gamma _e\\&\ne \mathbb {E}[X_1]{\mathbb {E}}[X_2-X_1]=(-\gamma _e)\times 1=-\gamma _e. \end{aligned}$$

## Next order asymptotics of optimal $k^*$

We now derive the next order asymptotics of the optimal $k^*$ derived in Sect. 5. Recall from (50) that $k^*\sim \alpha =p(1+W)\sim \log N$ as $N\rightarrow \infty$ where $W=W_0(z)$ and $z=(C/p)(AN)^{1/p}$ for p>0. Setting L=logz, the LambertW function has the following large argument expansion (Corless et al. 1996),

53 $$\begin{aligned} W_0(z) =L-\log L+O\Big (\frac{\log L}{L}\Big )\quad \text {as }L\rightarrow \infty . \end{aligned}$$

Since

$$\begin{aligned}&L=\log z=\log ((C/p)(AN)^{1/p}) =\frac{1}{p}\log N+\frac{1}{p}\log A+\log (C/p)\\&\quad \sim \frac{1}{p}\log N\quad \text {as }N\rightarrow \infty , \end{aligned}$$

we have that

$$\begin{aligned} \log L =\log \log N-\log p +O\Big (\frac{1}{\log N}\Big )\quad \text {as }N\rightarrow \infty . \end{aligned}$$

Combining this with (53) yields

54 $$\begin{aligned} \alpha =p(1+W) =\log N-p\log \log N+p+\log A+p\log C+o(1)\quad \text {as }N\rightarrow \infty , \end{aligned}$$

which, after combining with the relation $k^*=\alpha +\log k^*-3+1/(\alpha +\log k^*-1)$ in (49), yields

$$\begin{aligned} k^*=\log N +(1-p)\log \log N +[p+\log A+p\log C-3]+o(1)\quad \text {as }N\rightarrow \infty . \end{aligned}$$

It is straightforward to check that this estimate is self-consistent with (49). Hence, the geometry-dependent constants *A* and *C* affect the optimal $k^*$ through an additive constant. For the case of a circular cell with Dirichlet boundary conditions, the constant in (36) ($\kappa =\infty$) yields

$$\begin{aligned} k^*_{\text {Dir}} =\log N +\tfrac{1}{2}\log \log N-\tfrac{1}{2}\log (\pi R)-5/2+o(1)\quad \text {as }N\rightarrow \infty . \end{aligned}$$

Notice that all of the factors of the diffusivity *D* have canceled. Hence, in accord with the rest of our results, the diffusivity controls the timescale of when particles arrive, but it does not affect the optimal number to use for source inference. Notice also that farther sources (larger *R*) decreases the number of arrivals worth retaining, which has the interpretation that directional information degrades faster with respect to the ordered arrivals for more distant sources.
